# Sequence variability of the respiratory syncytial virus (RSV) fusion gene among contemporary and historical genotypes of RSV/A and RSV/B

**DOI:** 10.1371/journal.pone.0175792

**Published:** 2017-04-17

**Authors:** Anne M. Hause, David M. Henke, Vasanthi Avadhanula, Chad A. Shaw, Lorena I. Tapia, Pedro A. Piedra

**Affiliations:** 1 Department of Molecular Virology and Microbiology, Baylor College of Medicine, Houston, Texas, United States of America; 2 Department of Translational Biology and Molecular Medicine, Baylor College of Medicine, Houston, Texas, United States of America; 3 Department of Molecular and Human Genetics, Baylor College of Medicine, Houston, Texas, United States of America; 4 Department of Pediatrics and Pediatric Surgery, Universidad de Chile, Santiago, Chile; 5 Virology Program, Institute of Biomedical Sciences (ICBM), Facultad de Medicina, Universidad de Chile, Santiago, Chile; 6 Department of Pediatrics, Baylor College of Medicine, Houston, Texas, United States of America; Imperial College London, UNITED KINGDOM

## Abstract

**Background:**

The fusion (F) protein of RSV is the major vaccine target. This protein undergoes a conformational change from pre-fusion to post-fusion. Both conformations share antigenic sites II and IV. Pre-fusion F has unique antigenic sites p27, ø, α2α3β3β4, and MPE8; whereas, post-fusion F has unique antigenic site I. Our objective was to determine the antigenic variability for RSV/A and RSV/B isolates from contemporary and historical genotypes compared to a historical RSV/A strain.

**Methods:**

The F sequences of isolates from GenBank, Houston, and Chile (N = 1,090) were used for this analysis. Sequences were compared pair-wise to a reference sequence, a historical RSV/A *Long* strain. Variability (calculated as %) was defined as changes at each amino acid (aa) position when compared to the reference sequence. Only aa at antigenic sites with variability ≥5% were reported.

**Results:**

A total of 1,090 sequences (822 RSV/A and 268 RSV/B) were analyzed. When compared to the reference F, those domains with the greatest number of non-synonymous changes included the signal peptide, p27, heptad repeat domain 2, antigenic site ø, and the transmembrane domain. RSV/A subgroup had 7 aa changes in the antigenic sites: site I (N = 1), II (N = 1), p27 (N = 4), α2α3β3β4(AM14) (N = 1), ranging in frequency from 7–91%. In comparison, RSV/B had 19 aa changes in antigenic sites: I (N = 3), II (N = 1), p27 (N = 9), ø (N = 4), α2α3β3β4(AM14) (N = 1), and MPE8 (N = 1), ranging in frequency from 79–100%.

**Discussion:**

Although antigenic sites of RSV F are generally well conserved, differences are observed when comparing the two subgroups to the reference RSV/A *Long* strain. Further, these discrepancies are accented in the antigenic sites in pre-fusion F of RSV/B isolates, often occurring with a frequency of 100%. This could be of importance if a monovalent F protein from the historical GA1 genotype of RSV/A is used for vaccine development.

## Background

Respiratory syncytial virus (RSV) is a major cause of lower respiratory tract illness (LRTI) among infants and young children and contributes significantly to morbidity and mortality in this age group. RSV is classified into two subgroups, RSV/A and RSV/B, based on variation in the attachment (G) gene. Viruses from both subgroups circulate, though usually one subgroup dominates a given RSV season [1]. The G protein and fusion (F) protein are the only two surface glycoproteins capable of inducing a neutralizing antibody response [2]. However, the F protein is far more conserved than the G protein and, for this reason, has been the major antigen of focus for RSV vaccine development [3]. There is currently no licensed vaccine against RSV; however, there is a large pipeline containing candidate vaccines that are in preclinical to late stages of development [4]. Most of these vaccines are monovalent and utilize the F protein or sequence isolated in the 1960s from an RSV/A virus belonging to the GA1 genotype.

The RSV/A and RSV/B subgroups are further divided into genotypes based on variability in the distal third of the G gene, the hypervariable mucin-like domain [1,5]. During RSV season more than one genotype from the same RSV subgroup co-circulates within a community outbreak. The GA2 genotype has been the dominant genotype for RSV/A for nearly a decade. However, it is rapidly being replaced by the Ontario (ON1) genotype [6]. The Buenos Aires (BA) genotype has been the dominant genotype for RSV/B since 2005 [7]. Interestingly, both ON1 and BA have a unique duplication in the distal third of their G genes, 72 and 60 nucleotides respectively [7,8].

The F protein has been identified as having at least two dominant conformations: the pre-fusion and post-fusion F forms. The F protein’s pre-fusion conformation is metastable and readily rearranges into the stable post-fusion conformation [9]. Each of these conformations has been expressed as a protein crystal; however, modifications had to be made to stabilize the F protein, in particular, for the pre-fusion conformation. Thus it is possible that the pre-fusion protein crystallization may not represent the protein’s true form prior to virus-to-cell fusion. Both the pre-fusion and post-fusion conformation of the F protein are being explored as vaccine candidates [4,10,11]. These two conformations share some antigenic sites but also have their own antigenic sties. Two known antigenic sites (II and IV) are present in the pre- and post-fusion F [12,13]. Antigenic site II is the targeted site of the therapeutic monoclonal antibody, palivizumab. In addition, pre-fusion F has antigenic site ø, MPE-8, α2, α3, β3 & β4 (recognized by AM14), and p27 [9,14–16]; post-fusion F has the unique antigenic site I [17].

Although the F protein is generally thought to be well conserved, variability in some of the F domains has been observed in the signal peptide, transmembrane domain, not defined 2 site, and antigenic site ø [18]. In this report we examine the sequence variability of the F gene from a large bank of RSV sequences that span over 50 years. To better understand the impact this variability may have on vaccine development, we have focused on the antigenic sites of the pre-fusion and post-fusion F and used as our reference the F gene from a historical sequence. This reference gene belongs to the genotype GA1 which is often utilized in the development of RSV vaccines.

## Materials and methods

### Virus strains

In order to robustly represent and categorize the contemporary virus, previously sequenced and published RSV clinical isolates from the Department of Molecular Virology and Microbiology of Baylor College of Medicine, Houston, Texas (n = 118) and the Programa de Virología of Universidad de Chile (n = 102) were utilized in this study [18]. An additional 1017 RSV F gene sequences were obtained from the GenBank (www.ncbi.nlm.nih.gov/genbank/) database during October 2015 (S1 Table). GenBank sequences represent the publicly available sequence information for the RSV F gene provided by multiple study sites from 1961 through 2014. This data is not longitudinal surveillance information and should not be interpreted as such. All available RSV F gene sequences at the time of the download were considered in this study. When available, the corresponding G gene was also acquired and included. Additional information on the sequences, including date of sample collection and country of origin, was also obtained when such information was available.

### Genotype assignment

To ensure the historical data’s validity for every sequence, an unstructured cluster analysis of viral subgroup was conducted. Based on pairwise similarity scoring between any two viral sequences, the Lance-Williams dissimilarity score was used to create major group populations within the entire population of viral sequences. This method of categorizing the viral subgrouping was conducted among all F sequences and the two major groupings of G sequences (those with the duplication of the distal third of the G gene and those without). Once two major groups (representing RSV/A and RSV/B) had been established, the cluster split was compared to a priori subgroup information. Only strains which grouped the same between the a priori (historical call) and unstructured genotype call were utilized. In addition, sequences deemed of poor fidelity were removed. Between these control steps, 147 F sequences were removed from the analysis (S1 Fig).

To understand different populations of RSV, similar viral genome sequences are catagorized into genotypes. This assignment was preferentially performed on the virus’ G gene then its F gene. As per convention, the distal third of the gene was utilized for genotype assignment [1]. This region of sequence was selected by multiply aligning all G gene sequences then removing the region from the 649^th^ nucleotide to the 5’ end, with respect to the reference sequence. The remaining sequence represents ~27.7% of the gene. The surrounding sequence was seen to be relatively conserved between strains and provided a buffer before the insertion position seen in BA and ON genotypes. For those sequences without a corresponding G gene, only the F gene was used to provide genotype assignment as previously described [18]. By comparing every strain’s gene to everyother’s, we were able to rank the similarity of strains independently of one another. Based on this ranking we were able to group previously unassigned viral strains to their most appropriate genotype according to the similarity scores. Basic assumptions were acted on previous to the assignment of the similarity scores, creating informed distinct groupings. These assumptions distinguish obvious viral classifications, i.e. subgroups A vs. B and those genotypes which contain duplications within the distal third of the G gene, genotypes BA and ON.

Our similarity score ranking is based off of the maximal pair-wise similarity score of the multiply aligned genes (i.e. nearest neighbor method), encompassing both the non-genotyped laceled sequences and previously-genotype-labeled reference sequences was used in genotype assignment. Pair-wise alignment was conducted on DNA under a Smith-Waterman algorithm implemented using R version 3.0.1 (Biostring package version 2.30.1). A substitution matrix of 2 and -2 for matches and mismatches, respectively, was used, along with a -6 and -0.2 penalty for gap openings and extensions, respectively. RSV subgroups were quarantined during genotype assignment. G gene scoring was conducted only for non-genotyped sequences not seen with the insertion (exclusion of Ontario and Buenos Ares genotypes). Known genotypes were taken *a priori* and then amended using the insertion within the distal third of the G gene to assign genotypes to those isolates with duplications in the distal third of the G gene. Only scores of known genotype sequences were utilized as references for a maximal similarity to the unknown counterparts.

To ensure all sequences were aligned correctly, the conservative glycosylation sites were confirmed to have 100% consensus. Additional steps were taken to ensure accurate genotype assignment. First, the sequence length of the G gene was examined to ensure that those sequences with insertions were correctly assigned to their respective genotypes (ON1 or BA). Next, phylogenetic trees for the F and G gene were constructed to examine genotype clustering. Tree construction was conducted in a bootstrap fashion using 1,000 iterations. The optimized trees allowed for topology, base frequencies, the rate matrix, and the proportion of variable size to get optimized. Parameters were chosen by maximizing the tree’s log likelihood of the protein. Tree construction was conducted in R 3.3.0 (under the phangorn package v. 2.0.4).

### Amino acid variability analysis by subgroup and genotype

A final goal was to characterize the stability of the RSV F protein. This was accomplished by viewing changes within the F protein. To determine the amino acid variability in the F domains of RSV/A and RSV/B, the RSV/A and RSV/B subgroups were compared to the historical RSV/A *Long* strain (ATCC VR-26; RSV/A Long), a GA1 genotype.

As the specific type of nucleic acid change is informative, amino acids with both synonomous and nonsynonomous nucleotide changes were considered for our analysis. When constructing the nonsynonymous/synonomous bar chart of the amino acids comprising F gene, all sequences were grouped by distinct subgroup. Each sequence contributed equal influence to the graph. Codons with an unknown or missing base were dropped from the analysis. Previously defined F gene domains were assigned color blocks [19,20]. Additionally, antigenic sites were highlighted in shades of gray respective to the F protein formation on which they are found (pre-fusion, post-fusion, or both).

Variability was reported as the percentage of each unique amino acid at a given residue found in a subgroup. Individual genotypes of each subgroup contributed equally to the proportion of amino acids found at each residue. Genotypes with fewer than five sequences were excluded from the analysis. Changes occurring at ≤5% variability were excluded from this report.

Amino acid variability was also decomposed by genotype. Within genotype differences from the reference sequence were reported as the percentage of each unique amino acid found in a genotype at a given residue. All changes were reported for genotypes [1,21–25].

### Entropy analysis

In order to further quantify sequence variability, each amino acid and nucleotide position of the F protein was examined using an estimate of Shannon (information) entropy, defined as ∑_*i*_−*i* log(*i*), where *i* is the weighted proportion of each unique amino acid found in a given population and at a given residue. This measure of variability is representative of the disorder of each amino acid position within its populations (subgroups RSV/A and RSV/B). Thus, entropy is minimized when perfect consensus is found at a position; it is maximized when there is a uniform distribution over all options. Individual genotypes of each subgroup contributed equally to the proportion of amino acids found at a given residue.

## Results

A total of 1,090 RSV F gene sequences were utilized for this study. Of these, 352 had been previously assigned a genotype and were used as references for genotype assignment and construction of phylogenetic trees. The remaining 873 were assigned genotypes based on our previously described methods. Of the 586 with a corresponding G gene, 90 were observed to have a duplication in the distal third of the G gene indicative of the genotypes ON (72 nucleotide insertion) or BA (60 nucleotide insertion). The remaining 496 sequences with a corresponding G gene were genotyped based on the distal third of the G gene. Those 288 sequences without a corresponding G gene were genotyped based on pair wise assignment of the full F gene.

Of the 1,090 sequences, 822 were from the RSV/A subgroup and 268 were RSV/B (Table 1). Among the RSV/A subgroup, the most dominant genotypes were GA2 (44%) and GA5 (36%). The most dominant genotype among the RSV/B subgroup was BA (71%). Sequences of a particular genotype generally clustered together on the phylogenetic trees of their respective subgroups. Distance between branches of the phylogenetic trees was greater among the G trees (S2 Fig, S3 Fig) than the F trees (S4 Fig, S5 Fig), indicating greater variability among the G sequences than the F sequences.

**Table 1:** Number of sequences from GenBank, Houston, and Chile grouped by their pairwise similarity score assigned genotypes among the RSV/A and RSV/B subgroups.

Genotypes		Number of Sequences
**RSV/A**	GA1	38
	GA5	294
	GA3	10
	GA4	2
	GA7	13
	NA1	13
	SAA	5
	GA2	364
	ON	83
	*RSV/A SUB-TOTAL*	**822**
**RSV/B**	GB1	12
	GB4	16
	SAB	12
	GB3	38
	BA	190
	*RSV/B SUB-TOTAL*	**268**
**TOTAL**		**1,090**

**Fig 1:**
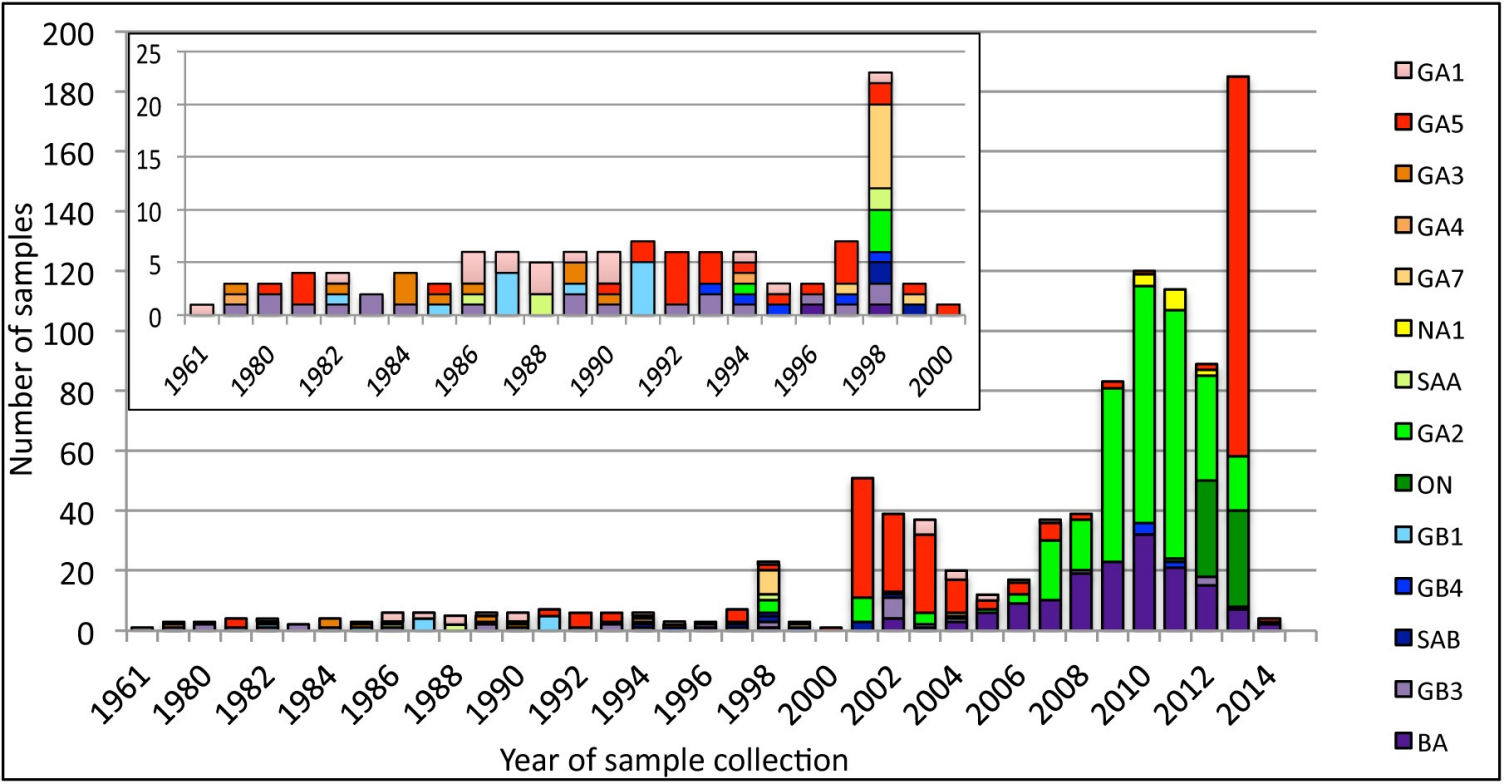
Appearance of RSV/A and RSV/B genotypes and dominance over time (1961–2014). Sequences assigned genotypes were assessed by their sample acquisition date. The included inset depicts those years (1961–2000) with a small number of available sequences.

Descriptive information was available for 965 (86%) F gene sequences, including date of sample collection. This information is not longitudinal and therefore not necessarily representative of the epidemic in natural populations. The oldest sample included in this dataset is from 1956, the majority of the samples were obtained between 2001 and 2014. The appearance of different genotypes and shifts in their dominance are evident when the genotype assignments are plotted by year the samples were obtained (Fig 1). It is interesting to note that there was a resurgence of GA5 viruses in 2013. It is also clear that, although RSV/A predominates in most years, there is annual co-circulation of the two subgroups.

### Amino acid variability of RSV subgroups

**Fig 2:**
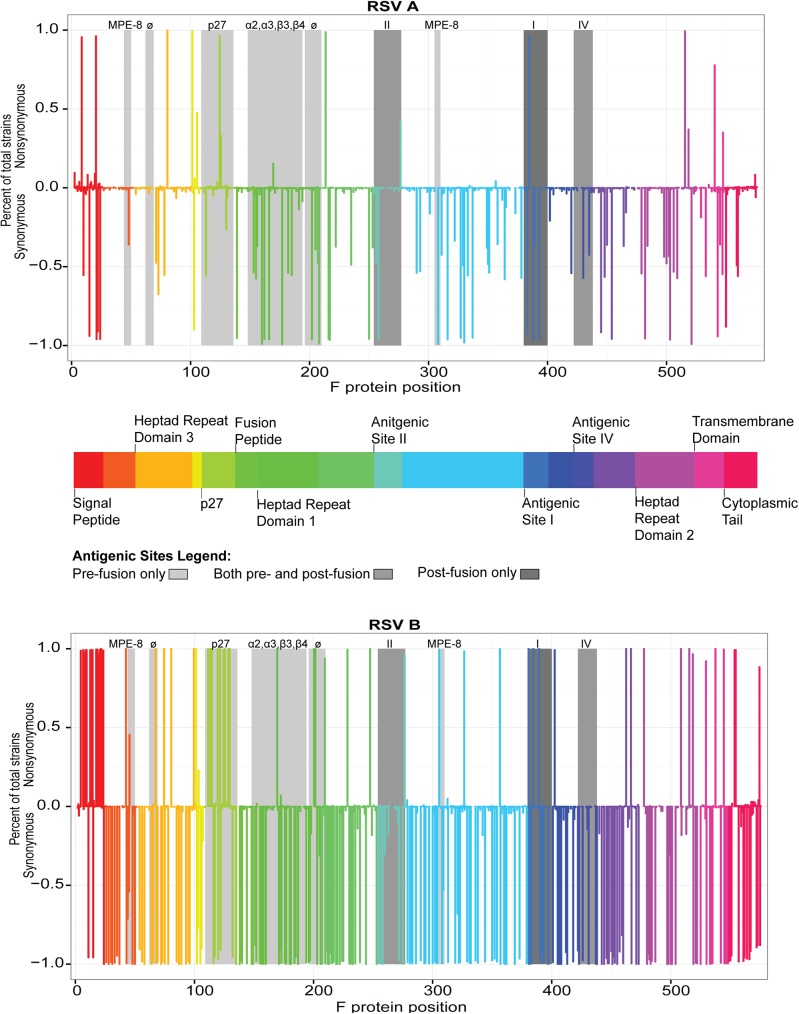
The fusion genes of RSV/A isolates are more similar to the RSV/A *Long* strain than RSV/B isolates. Non-synonymous/synonymous ratio graph of amino acids with non-synonymous or synonomous changes in the fusion gene for a) RSV/A isolates and b) RSV/B isolates (compared to the RSV/A *Long* strain). Fusion gene domains are depicted by assigned color blocks. Antigenic sites are highlighted in shades of gray respective to the protein conformation on which they are found.

The F domains of RSV/A and RSV/B were compared to the historical RSV/A *Long* strain (ATCC VR-26; RSV/A Long). When compared to the RSV/A *Long* strain, viruses in the RSV/A subgroup had a number of nucleotide changes, the majority of which resulted in synonymous amino acid changes (Fig 2). Those domains with the greatest number of non-synonymous changes included the signal peptide, p27, heptad repeat domain 2, antigenic site ø and the transmembrane domain. Shown in S2 Table are all the non-synomynous changes that were detected in domains that have not been reported to have antigenic sites. Overall, there were a greater number of non-synonymous nucleotide changes in the RSV/B subgroup (N = 60) than the RSV/A subgroup (N = 21), when compared to the RSV/A *Long* strain. For both subgroups, approximately one-third of the non-synonmyous changes occurred in antigenic sites. Those domains that had non-synonymous changes among RSV/A also had non-synonymous changes among RSV/B, and often occurred in the same amino acid residue. However the changes were more numerous among RSV/B and occurred with a higher frequency. For example, the signal peptide of RSV/B had 15 amino acid changes all occurring with a frequency of >90%, with the exception of a secondary change in AA4 that occurred in 7% of sequences. Conversly, the signal peptide of RSV/A had four amino acid changes, none of which occurred at a frequency >90%. A number of non-synonymous changes among RSV/B were seen in other additional domains, including antigenic sites.

The viruses in the RSV/A subgroup have fewer amino acid changes in antigenic sites than the viruses in the RSV/B subgroup when compared to the historical RSV/A *Long* strain (Table 2). Those seven amino acid changes in antigenic sites in RSV/A (with frequency >5%) occurred in sites I, II, p27, and α2α3β3β4 (AM14) and ranged from 7–91% in frequency. A total of nineteen amino acid changes occurred in the antigenic sites of RSV/B viruses. The majority of these changes (N = 15) occurred in pre-fusion antigenic sites (antigenic site ø, MPE-8, α2α3β3β4 (AM14), and p27) and ranged from 79–100% in frequency. Seven amino acids (384, 276, 124, 125, 129, 129, and 169) among the antigenic sites share vulnerability to change in both RSV/A and RSV/B isolates. Changes occurred at a greater rate in RSV/B. For example, a single change occurred in site p27 of RSV/A, L129V, at a rate of 14%. Among the RSV/B subgroup, a change occured at the same amino acid site. This change from leucine to isoleucine at a rate of 100%.

**Table 2:** Frequency of amino acid changes in antigenic sites for of the fusion gene RSV/A (N = 822) and RSV/B (N = 268) compared to the RSV/A *Long* strain.

Antigenic Site	Amino Acids	RSV/A		RSV/B	
		AA Change	Frequency	AA Change	Frequency
**I**	380–400	-	-	N380S	100%
		V384I	91%	V384T	100%
		-	-	P389S	100%

**II**	254–277	N276S	38%	N276S	100%

**IV**	422–438	-	-	-	-

**p27**	109–136	-	-	L111A	100%
		-	-	R113Q	100%
		-	-	F114Y	98%
		-	-	L119I	100%
		-	-	N121T	100%
		T122A	9%	-	-
		K124N	87%	K124N	100%
		T125N	16%	T125L	98%
		-	-	T128S	100%
		L129V	14%	L129I	100%

**ø**	62–69, 196–210	-	-	N67T	100%
		-	-	D200N	100%
		-	-	K201N	100%
		-	-	K209Q	88%

**α2α3β3β4(AM14)**	148–194	S169N	7%	S169N	100%

**MPE8**	44–50, 305–310	-	-	L45F	79%

Changes with ≤5% frequency were omitted from this table. Individual genotypes of each subgroup contributed equally to the proportion of amino acids found at each residue.

### Amino acid variability among RSV genotypes

The F antigenic sites of isolates for each genotype were compared to the historical RSV/A *Long* strain (ATCC VR-26; RSV/A Long). Among antigenic site I (Table 3), the change V384I was observed at a rate of ≥90% for all RSV/A genotypes except GA1, for which the change was observed with less frequency (50%). The change P389S was observed among GA2 strains at a very low rate (0.30%) and among all RSV/B genotypes with a frequency of 100%. An additional amino acid change, V384T, was observed among all RSV/B genotypes with a rate of 100%.

**Table 3:** Genotype specific amino acid changes when compared to the RSV/A *Long* strain in antigenic site I.

** **	**380**	**381**	**382**	**383**	**384**	**385**	**386**	**387**	**388**	**389**	**390**	**391**	**392**	**393**	**394**	**395**	**396**	**397**	**398**	**399**	**400**
	N	L	C	N	V	D	I	F	N	P	K	Y	D	C	K	I	M	T	S	K	T
**GA1**	.	.	.	.	I^b^	.	.	.	.	.	.	.	.	.	.	.	.	.	.	.	.
2° AA					.^b^																
**GA5**	.	.	.	.	I^a^	.	.	.	.	.^a^	.	.	.	.	.	.	.	.	.	.^a^	.^a^
2° AA					.^d^															I^d^	A^d^
**GA3**	.	.	.	.	I^a^	.	.	.	.	.	.	.	.	.	.	.	.	.	.	.	.
**GA7**	.	.	.	.	I^a^	.	.	.	.	.	.	.	.	.	.	.	.	.	.	.	.
2° AA					V^c^																
**NA1**	.	.	.	.	I^a^	.	.	.	.	.	.	.	.	.	.	.	.	.	.	.	.
**SAA**	.	.	.	.	I^a^	.	.	.	.	.	.	.	.	.	.	.	.	.	.	.	.
**GA2**	.^a^	.^a^	.	.	I^a^	.	.	.	.	.^a^	.^a^	.	.	.	.	.	.	.	.	.	.
2° AA	D^d^	F^d^			.					S^d^	N^d^										
3° AA					T^d^																
**ON**	.	.^a^	.	.	I^a^	.	.	.	.	.	.	.	.	.	.	.	.	.	.	.	.
2° AA		F^d^																			
	**380**	**381**	**382**	**383**	**384**	**385**	**386**	**387**	**388**	**389**	**390**	**391**	**392**	**393**	**394**	**395**	**396**	**397**	**398**	**399**	**400**
	N	L	C	N	V	D	I	F	N	P	K	Y	D	C	K	I	M	T	S	K	T
**GB1**	S^a^	.	.	.	T^a^	.	.	.	.	S^a^	.	.	.	.	.	.	.	.	.	.	.
**GB4**	S^a^	.	.	.	T^a^	.	.	.	.	S^a^	.	.	.	.	.	.	.	.	.	.	.
**SAB**	S^a^	.	.	.	T^a^	.	.	.	.	S^a^	.	.	.	.	.	.	.	.	.	.	.
**GB3**	S^a^	.	.	.	T^a^	.	.	.	.	S^a^	.	.	.	.	.	.	.	.	.	.	.
**BA**	S^a^	.	.	.	T^a^	.	.	.	.	S^a^	.	.	.	.	.	.	.	.	.	.	.

The percentage of each unique amino acid found in a genotype at a given residue is indicated by the superscripts:

^d^(≤1%)

^c^(2–45%)

^b^(46–89%), and

^a^(≥90%).

Among antigenic site II (Table 4), the amino acid change N276S was observed among contemporary RSV/A genotypes GA2, NA1, and ON, occurring at rate of 70%, 100%, and 99%, respectively. This change occurred in all RSV/B genotypes with a frequency of 100%, with the exception of BA, for which the change occurred in 95% of sequences.

**Table 4:** Genotype specific amino acid changes when compared to the RSV/A *Long* strain in antigenic site II.

** **	**254**	**255**	**256**	**257**	**258**	**259**	**260**	**261**	**262**	**263**	**264**	**265**	**266**	**267**	**268**	**269**	**270**	**271**	**272**	**273**	**274**	**275**	**276**	**277**
	N	S	E	L	L	S	L	I	N	D	M	P	I	T	N	D	Q	K	K	L	M	S	N	N
**GA1**	.	.	.	.	.	.	.	.	.	.	.	.	.	.	.	.	.	.	.	.	.	.	.	.
**GA5**	.	.	.	.	.	.	.	.	.	.	.	.	.	.	.^a^	.	.	.	.	.	.	.	.	.
2° AA															I^d^									
**GA3**	.	.	.	.	.	.	.	.	.	.	.	.	.	.	.	.	.	.	.	.	.	.	.	.
**GA7**	.	.	.	.	.	.	.	.	.	.	.	.	.	.	.	.	.	.	.	.	.	.	.	.
**NA1**	.	.	.	.	.	.	.	.	.	.	.	.	.	.	.	.	.	.	.	.	.	.	S^a^	.
**SAA**	.	.	.	.	.	.	.	.	.	.	.	.	.	.	.	.	.	.	.	.	.	.	.	.
**GA2**	.	.^a^	.	.	.	.	.	.	.	.	.	.	.	.	.	.	.	.	.	.	.	.	S^b^	.
2°AA		N^d^																					.^c^	
**ON**	.	.^a^	.	.	.	.	.	.	.	.	.	.	.	.	.	.	.	.	.	.	.	.	S^a^	.
2° AA		G^c^																					.	
** **	**254**	**255**	**256**	**257**	**258**	**259**	**260**	**261**	**262**	**263**	**264**	**265**	**266**	**267**	**268**	**269**	**270**	**271**	**272**	**273**	**274**	**275**	**276**	**277**
	N	S	E	L	L	S	L	I	N	D	M	P	I	T	N	D	Q	K	K	L	M	S	N	N
**GB1**	.	.	.	.	.	.	.	.	.	.	.	.	.	.	.	.	.	.	.	.	.	.	S^a^	.
**GB4**	.	.	.	.	.	.^a^	.	.	.	.	.	.	.	.	.	.	.	.	.	.	.	.	S^a^	.
2° AA						T^c^																		
**SAB**	.	.	.	.	.	.	.	.	.	.	.	.	.	.	.	.	.	.	.	.	.	.	S^a^	.
**GB3**	.	.^a^	.	.	.	.	.	.	.	.	.	.	.	.	.	.	.	.	.	.	.	.	S^a^	.
2° AA		G^c^																						
**BA**	.	.	.	.	.	.	.	.	.	.	.	.	.	.	.	.	.	.	.	.	.	.	S^a^	.
2° AA																							.^c^	

The percentage of each unique amino acid found in a genotype at a given residue is indicated by the superscripts:

^d^(≤1%)

^c^(2–45%)

^b^(46–89%), and

^a^(≥90%).

Antigenic site IV (Table 5) was well conserved among all genotypes of RSV/A and RSV/B. Two amino acid changes were observed at a low frequency (≤1%) among the GA5 genotype.

**Table 5:** Genotype specific amino acid changes when compared to the RSV/A *Long* strain in antigenic site IV.

** **	**422**	**423**	**424**	**425**	**426**	**427**	**428**	**429**	**430**	**431**	**432**	**433**	**434**	**435**	**436**	**437**	**438**
	C	T	A	S	N	K	N	R	G	I	I	K	T	F	S	N	G
**GA1**	.	.	.	.	.	.	.	.	.	.	.	.	.	.	.	.	.
**GA5**	.	.	.	.	.	.	.^a^	.	.	.	.	.	.	.	.^a^	.	.
2° AA							D^d^								F^d^		
**GA3**	.	.	.	.	.	.	.	.	.	.	.	.	.	.	.	.	.
**GA7**	.	.	.	.	.	.	.	.	.	.	.	.	.	.	.	.	.
**NA1**	.	.	.	.	.	.	.	.	.	.	.	.	.	.	.	.	.
**SAA**	.	.	.	.	.	.	.	.	.	.	.	.	.	.	.	.	.
**GA2**	.	.	.	.	.	.	.	.	.	.	.	.	.	.	.	.	.
**ON**	.	.	.	.	.	.	.	.	.	.	.	.	.	.	.	.	.
** **	**422**	**423**	**424**	**425**	**426**	**427**	**428**	**429**	**430**	**431**	**432**	**433**	**434**	**435**	**436**	**437**	**438**
	C	T	A	S	N	K	N	R	G	I	I	K	T	F	S	N	G
**GB1**	.	.	.	.	.	.	.	.	.	.	.	.	.	.	.	.	.
**GB4**	.	.	.	.	.	.	.	.	.	.	.	.	.	.	.	.	.
**SAB**	.	.	.	.	.	.	.	.	.	.	.	.	.	.	.	.	.
**GB3**	.	.	.	.	.	.	.	.	.	.	.	.	.	.	.	.	.
**BA**	.	.	.	.	.	.	.	.	.	.	.	.	.	.	.	.	.

The percentage of each unique amino acid found in a genotype at a given residue is indicated by the superscripts:

^d^(≤1%)

^c^(2–45%)

^b^(46–89%), and

^a^(≥90%).

Site p27 was the most variable antigenic site (Table 6). The change K124N was observed at a rate of ≥90% for all RSV/A genotypes except GA1, for which the change was observed with less frequency (18%). Several amino acid changes occurred with high frequency among single RSV/A genotypes, including T122A in GA1 (61%), T125N in GA5 (89%), and L129V in GA7 (100%). Among the RSV/B genotypes, the following changes occurred with an observed frequency of ≥90%: L111A, R113Q, F114Y, L119I, N121T, K124N, T125L, T128S, L129I. These changes were also observed in the contemporary genotypes of RSV/A (GA2 and ON).

**Table 6:** Genotype specific amino acid changes when compared to the RSV/A *Long* strain in antigenic site p27.

** **	**109**	**110**	**111**	**112**	**113**	**114**	**115**	**116**	**117**	**118**	**119**	**120**	**121**	**122**	**123**	**124**	**125**	**126**	**127**	**128**	**129**	**130**	**131**	**132**	**133**	**134**	**135**	**136**
	R	E	L	P	R	F	M	N	Y	T	L	N	N	T	K	K	T	N	V	T	L	S	K	K	R	K	R	R
**GA1**	.	.	.	.	.	.^a^	.	.	.	.	.	.	.	A^b^	.	.^b^	.^b^	.	.	.	.	.	.	.	.	.	.	.
2° AA						V^c^								.^c^		N^c^	N^c^											
3° AA														P^c^		T^c^												
**GA5**	.	.	.	.	.	.^a^	.	.^a^	.	.	.	.	.^a^	.^a^	.	N^a^	N^a^	.	.^a^	.	.^a^	.	.^a^	.	.	.	.	.
2° AA						L^d^		Y^d^					S^d^	N^d^			.		I^c^		V^d^		N^d^					
**GA3**	.	.	.	.^a^	.	.	.	.	.^a^	.	.	.	.	.	.	N^a^	.	.	.	.	.	.	.	.	.	.	.	.
2° AA				S^c^					H^c^																			
**GA7**	.	.	.	.	.	.	.	.	.	.	.	.	.	.	.	N^a^	.	.	.	.	V^a^	.	.	.	.	.	.	.
**NA1**	.	.	.	.	.	.	.	.	.^a^	.	.	.	.	.	.	N^a^	.	.	.	.	.	.	.	.	.	.	.	.
2° AA									H^c^																			
**SAA**	.	.	.	.	.	.	.	.	.	.	.	.	.	.	.	N^a^	.	.	.	.	.	.	.	.	.	.	.	.
**GA2**	.	.	.	.	.	.^a^	.^a^	.^a^	.^a^	.^a^	.^a^	.^a^	.^a^	.^a^	.^a^	N^a^	.^a^	.	.^a^	.^a^	.	.^a^	.^a^	.^a^	.	.	.	.
2° AA						S^d^	R^d^	D^c^	H^c^	I^d^	I^d^	D^d^	S^d^	A^d^	N^d^	T^c^	I^d^		I^d^	I^d^		G^d^	R^d^	I^d^				
3° AA									N^d^		F^d^	K^d^		I^d^		Y^d^	A^d^											
**ON**	.	.	.	.	.	.	.	.	.	.	.	.^a^	.	.^a^	.	N^a^	.^a^	.	.^a^	.	.	.	.	.	.	.	.	.
2° AA												D^c^		A^c^		Y^d^	I^d^		I^d^									
3° AA																	N^d^											
	**109**	**110**	**111**	**112**	**113**	**114**	**115**	**116**	**117**	**118**	**119**	**120**	**121**	**122**	**123**	**124**	**125**	**126**	**127**	**128**	**129**	**130**	**131**	**132**	**133**	**134**	**135**	**136**
	R	E	L	P	R	F	M	N	Y	T	L	N	N	T	K	K	T	N	V	T	L	S	K	K	R	K	R	R
**GB1**	.	.	A^a^	.	Q^a^	Y^a^	.	.	.	.	I^a^	.	T^a^	.	.	N^a^	L^a^	.	.	S^a^	I^a^	.	.	.	.	.	.	.
**GB4**	.	.	A^a^	.	Q^a^	Y^a^	.	.	.	.	I^a^	.	T^a^	.	.	N^a^	L^a^	.	.	S^a^	I^a^	.	.	.	.	.	.	.
**SAB**	.	.	A^a^	.	Q^a^	Y^a^	.	.	.	.	I^a^	.	T^a^	.	.	N^a^	L^a^	.	.	S^a^	I^a^	.	.	.	.	.	.	.
**GB3**	.	.	A^a^	.	Q^a^	Y^a^	.^a^	.	.^a^	.	I^a^	.	T^a^	.	.	N^a^	L^a^	.	.	S^a^	I^a^	.	.	.	.	.	.	.
2° AA					H^c^	H^c^	T^c^		H^c^												T^c^							
**BA**	.	.	A^a^	.^a^	Q^a^	Y^a^	.^a^	.	.^a^	.	I^a^	.	T^a^	.^a^	.^a^	N^a^	L^a^	.	.^a^	S^a^	I^a^	.^a^	.	.	.	.	.	.
2° AA				S^d^			I^d^		C^d^				A^c^	I^d^	G^c^	S^c^	P^c^		A^c^		T^d^	I^d^						
3° AA													I^d^		R^d^	.^d^	S^d^											

The percentage of each unique amino acid found in a genotype at a given residue is indicated by the superscripts:

^d^(≤1%)

^c^(2–45%)

^b^(46–89%), and

^a^(≥90%).

Among antigenic site ø (Table 7), a number of amino acid changes occurred with low frequency within isolates of the RSV/A genotypes. Among RSV/B genotypes, the amino acid changes N67T, D200N, and K201N, occurred with a frequency of ≥90%. The amino acid change K209Q occurred more frequently in GB1 (100%), SAB (100%), BA (99.5%) genotypes than GB4 (63%) and GB3 (76%).

**Table 7:** Genotype specific amino acid changes when compared to the RSV/A Long strain in antigenic site ø.

** **	**62**	**63**	**64**	**65**	**66**	**67**	**68**	**69**	**196**	**197**	**198**	**199**	**200**	**201**	**202**	**203**	**204**	**205**	**206**	**207**	**208**	**209**	**210**
	S	N	I	K	E	N	K	C	K	N	Y	I	D	K	Q	L	L	P	I	V	N	K	Q
**GA1**	.^a^	.	.	.	.^a^	.	.	.	.	.	.	.	.	.	.	. ^a^	.	.	.	.	.	.	.
2° AA	R^c^				K^c^											V^c^							
**GA5**	.	.	.	.	.	.	.	.	.	.^a^	.	.	.	.	.	.	.	.	.	.	.	.	.
2° AA										T^d^													
**GA3**	.	.	.	.	.	.	.	.	.	.	.	.	.	.	.	.	.	.	.	.	.	.	.
**GA7**	.	.	.	.	.	.	.	.	.	.	.	.	.	.	.	.	.	.	.	.	.	.	.
**NA1**	.	.	.	.	.	.	.	.	.	.	.	.	.	.	.	.	.	.	.	.	.	.	.
**SAA**	.	.	.	.	.	.	.	.	.	.	.	.	.	.	.	.	.	.	.	.	.	.	.
**GA2**	.	.^a^	.	.	.	.	.	.	.	.	.	.^a^	.	.	.	.^a^	.	.	.^a^	.	.	.	.
2° AA		T^d^										M^d^				F^d^			V^d^				
**ON**	.	.	.	.	.	.	.	.	.	.	.	.	.	.	.	.	.	.	.	.	.	.	.
** **	**62**	**63**	**64**	**65**	**66**	**67**	**68**	**69**	**196**	**197**	**198**	**199**	**200**	**201**	**202**	**203**	**204**	**205**	**206**	**207**	**208**	**209**	**210**
	S	N	I	K	E	N	K	C	K	N	Y	I	D	K	Q	L	L	P	I	V	N	K	Q
**GB1**	.	.	.	.	.	T^a^	.	.	.	.	.	.	N^a^	N^a^	.	.	.	.	.	.	.	Q^a^	.
**GB4**	.	.	.	.	.	T^a^	.	.	.	.	.	.	N^a^	N^a^	.	.	.	.	.	.	.	Q^b^	.
2° AA																						.^c^	
**SAB**	.	.	.	.	.	T^a^	.	.	.	.	.	.	N^a^	N^a^	.	.	.	.	.	.	.	Q^a^	.
**GB3**	.	.	.	.^a^	.	T^a^	.^a^	.	.	.	.	.	N^a^	N^a^	.	.	.	.	.	.	.	Q^b^	.
2° AA				Q^c^		I^c^	N^c^															.^c^	
**BA**	.	.	.	.	.	T^a^	.	.	.	.^a^	.	.	N^a^	N^a^	.	.	.	.	.	.	.	Q^a^	.
2° AA						I^c^																.^d^	

The percentage of each unique amino acid found in a genotype at a given residue is indicated by the superscripts:

^d^(≤1%)

^c^(2–45%)

^b^(46–89%), and

^a^(≥90%).

Among site α2α3β3β4(AM14) (Table 8), a number of amino acid changes occurred at a low rate within the RSV/A genotypes. These changes include S169N, which was observed among GA1 (3%), GA5 (42%), and GA2 (0.5%). Among RSV/B genotypes, the amino acid change S169N was observed in all RSV/B genotypes at a frequency of 100%, except BA for which the change occurred in 99.5% of sequences.

**Table 8:** Genotype specific amino acid changes when compared to the RSV/A *Long* strain in antigenic site α2α3β3β4(AM14).

	**148**	**149**	**150**	**151**	**152**	**153**	**154**	**155**	**156**	**157**	**158**	**159**	**160**	**161**	**162**	**163**	**164**	**165**	**166**	**167**	**168**	**169**	**170**	**171**	**172**	**173**	**174**	**175**	**176**	**177**	**178**	**179**	**180**	**181**	**182**	**183**	**184**	**185**	**186**	**187**	**188**	**189**	**190**	**191**	**192**	**193**	**194**
	I	A	S	G	I	A	V	S	K	V	L	H	L	E	G	E	V	N	K	I	K	S	A	L	L	S	T	N	K	A	V	V	S	L	S	N	G	V	S	V	L	T	S	K	V	L	D
**GA1**	.^a^	.	.	.	.	.	.	.	.	.	.	.	.	.	.	.	.	.	.	.	.^a^	.^a^	.	.	.^a^	.^a^	.	.	.	.	.	.	.	.	.	.	.	.	.	.	.	.	.	.	.	.	.
2° AA	T^c^				V^c^																R^c^	N^c^			Q^c^	T^c^																					
3° AA					T^c^																																										
**GA5**	.	.	.	.	.	.	.	.	.	.	.	.	.	.	.	.	.	.^a^	.	.^a^	.	.^b^	.	.	.	.	.	.	.	.	.	.	.	.	.	.^a^	.	.	.	.	.	.	.	.	.	.	.
2° AA																		D^d^		L^c^		N^c^														T^d^											
**GA3**	.	.	.	.	.	.	.	.	.	.	.	.	.	.	.	.	.	.	.	.	.	.	.	.	.	.	.	.	.	.	.	.	.	.	.	.	.	.	.	.	.	.	.	.	.	.	.
**GA7**	.	.	.	.	.	.	.	.	.	.	.	.	.	.	.	.	.	.	.	.	.	.	.	.	.	.	.	.	.	.	.	.	.	.	.	.	.	.	.	.	.	.	.	.	.	.	.
**NA1**	.	.	.	.	.	.	.	.	.	.	.	.	.	.	.	.	.	.	.	.	.	.	.	.	.	.	.	.	.	.	.	.	.	.	.	.	.	.	.	.	.	.	.	.	.	.	.
**SAA**	.	.	.	.	.	.	.	.	.	.	.	.	.	.	.	.	.	.	.	.	.	.	.	.	.	.	.	.	.	.	.	.	.	.	.	.	.	.	.	.	.	.	.	.	.	.	.
**GA2**	.	.	.	.	.^a^	.	.	.	.	.	.	.	.	.	.	.	.	.^a^	.	.	.	.^a^	.	.	.	.	.	.	.	.	.	.	.	.^a^	.^a^	.^a^	.	.	.	.	.	.	.	.	.	.	.^a^
2° AA					T^d^													K^d^				N^d^												F^d^	I^d^	D^d^											H^d^
ON	.	.	.	.	.	.	.	.	.		.	.	.	.	.	.	.	.	.	.	.	.	.	.^a^	.	.	.	.	.	.	.	.	.	.	.	.	.	.	.	.	.	.	.	.	.	.	.
2° AA																								P^d^																							
** **	**148**	**149**	**150**	**151**	**152**	**153**	**154**	**155**	**156**	**157**	**158**	**159**	**160**	**161**	**162**	**163**	**164**	**165**	**166**	**167**	**168**	**169**	**170**	**171**	**172**	**173**	**174**	**175**	**176**	**177**	**178**	**179**	**180**	**181**	**182**	**183**	**184**	**185**	**186**	**187**	**188**	**189**	**190**	**191**	**192**	**193**	**194**
	I	A	S	G	I	A	V	S	K	V	L	H	L	E	G	E	V	N	K	I	K	S	A	L	L	S	T	N	K	A	V	V	S	L	S	N	G	V	S	V	L	T	S	K	V	L	D
**GB1**	.	.	.	.	.	.	.	.	.	.	.	.	.	.	.	.	.	.	.	.	.	N^a^	.	.	.	.	.	.	.	.	.	.	.	.	.	.	.	.	.	.	.	.	.	.	.	.	.
2° AA																															I																
**GB4**	.	.	.	.	.	.	.	.	.	.	.	.	.	.	.	.	.	.	.	.	.	N^a^	.	.	.	.	.	.	.	.	.	.	.	.	.	.	.	.	.	.	.	.	.	.	.	.	.
**SAB**	.	.	.	.	.	.	.	.	.	.	.	.	.	.	.	.	.	.	.	.	.	N^a^	.	.	.	.	.	.	.	.	.	.	.	.	.	.	.	.	.	.	.	.	.	.	.	.	.
**GB3**	.	.	.	.	.^a^	.	.	.	.	.	.	.	.	.	.	.	.	.	.	.	.	N^a^	.	.	.	.	.	.	.	.	.	.	.	.	.	.	.	.	.	.	.	.	.	.	.	.	.
2° AA					M^c^																																										
**BA**	.	.	.	.	.^a^	.	.	.	.	.	.	.	.	.	.	.	.	.	.	.	.	N^a^	.	.	.^a^	.^a^	.	.	.	.	.	.	.	.	.	.	.	.	.	.	.	.	.	.	.	.	.
2° AA					M^d^																	D^d^			Q^c^	A^d^																					
3° AA					V^d^																					L^d^																					

The percentage of each unique amino acid found in a genotype at a given residue is indicated by the superscripts:

^d^(≤1%)

^c^(2–45%)

^b^(46–89%), and

^a^(≥90%).

Among site MPE8 (Table 9), a number of amino acid changes occurred with low frequency within the RSV/A genotypes. The amino acid change L45F occurred with high frequency in all RSV/B genotypes GB1 (100%), GB4 (69%), SAB (100%), and GB3 (100%), except BA, for which the change occurred in 25% of sequences. The amino acid change L305I was observed at a rate of 100% for all RSV/B genotypes.

**Table 9:** Genotype specific amino acid changes when compared to the RSV/A *Long* strain in antigenic site MPE8.

** **	**44**	**45**	**46**	**47**	**48**	**49**	**50**	**305**	**306**	**307**	**308**	**309**	**310**
	Y	L	S	A	L	R	T	L	Y	G	V	I	D
**GA1**	.	.	.	.	.	.	.	.	.	.	.	.	.
**GA5**	.	.	.	.	.	.	.^a^	.	.	.	.	.	.
2° AA							I^d^						
**GA3**	.	.	.	.	.	.	.	.	.	.	.	.	.
**GA7**	.	.	.	.	.	.	.	.	.	.	.	.	.
**NA1**	.	.	.	.	.	.	.	.	.	.	.	.	.
**SAA**	.	.	.	.	.	.	.		.	.	.	.	.
**GA2**	.^a^	.	.	.	.	.	.	.	.	.	.	.^a^	.
2° AA	F^d^											M^d^	
**ON**	.	.	.	.	.	.	.	.	.	.	.	.	.
** **	**44**	**45**	**46**	**47**	**48**	**49**	**50**	**305**	**306**	**307**	**308**	**309**	**310**
	Y	L	S	A	L	R	T	L	Y	G	V	I	D
**GB1**	.	F^a^	.	.	.	.	.	I^a^	.	.	.	.	.
**GB4**	.	F^b^	.	.	.	.	.	I^a^	.	.	.	.	.
2° AA		.^c^											
**SAB**	.	F^a^	.	.	.	.	.	I^a^	.	.	.	.	.
**GB3**	.	F^a^	.	.	.	.	.	I^a^	.	.	.	.	.
**BA**	.	.^b^	.	.	.	.	.	I^a^	.	.	.	.	.
2° AA		F^c^											
3° AA		I^d^											

The percentage of each unique amino acid found in a genotype at a given residue is indicated by the superscripts:

^d^(≤1%)

^c^(2–45%)

^b^(46–89%), and

^a^(≥90%).

### Amino acid entropy of RSV subgroups

**Fig 3:**
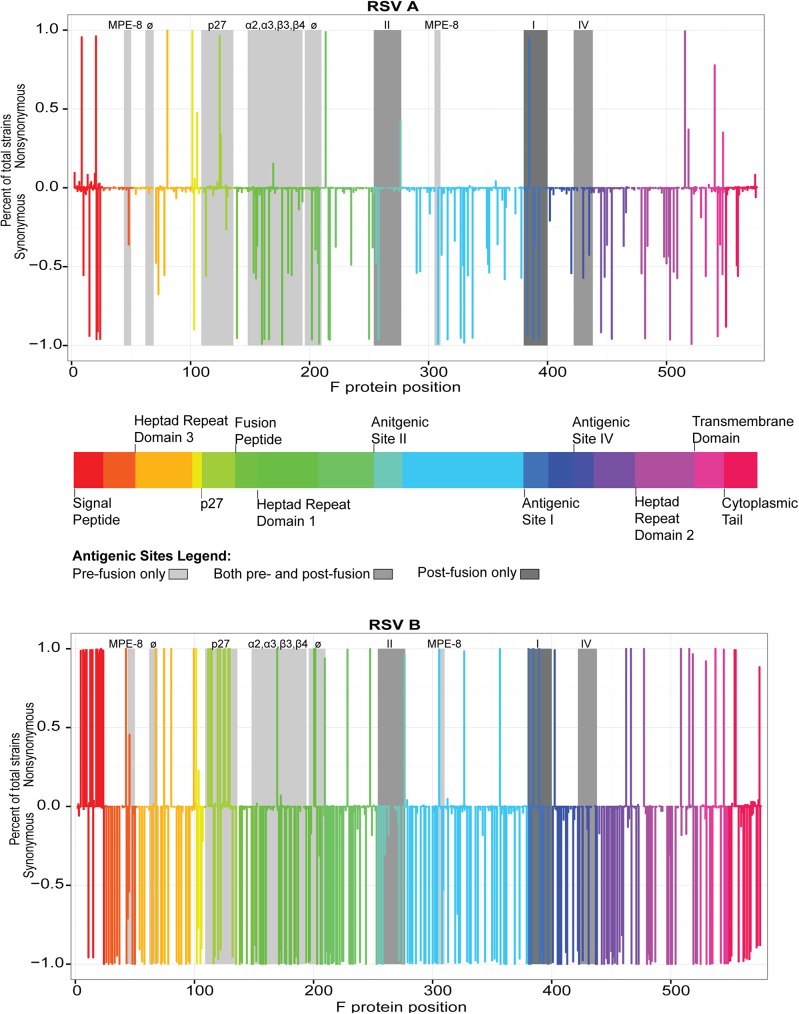
The entropy values in amino acids within antigenic sites of the fusion gene of RSV/A and RSV/B have a similar distribution. Entropy was defined as ∑_*i*_−*i* log(*i*). Individual genotypes of each subgroup contributed equally to the proportion of amino acids found at a given residue.

The amino acids of the F protein were analyzed for entropy, another measure for variability. The theoretical range for entropy is 0 to 3.3 (given that all amino acids have equal representation at any one particular location). Amino acids with an entropy values of 0.1 or less are considered stable. The higher the entropy value the greater the likelihood of variability at that residue. Those amino acids within the top 5% (≥95 percentile) of entropy are reported in Table 10. For RSV/A, the mean entropy value of the amino acids within the top 5% of entropy was 0.39 (0.18–1.03). For RSV/B, the mean entropy value of the top 5% was 0.26 (0.10–0.92). Many of these amino acids that fell within the top 5% of entropy belonged to the signal peptide, cytoplasmic tail, transmembrane domain, or p27. In addition, a number of these amino acids with the highest entropy values were within antigenic sites. Among RSV/A isolates, the amino acids of antigenic sites with high entropy values resided in antigenic sites I and II, p27, α2α3β3β4(AM14), while they were found in MPE-8, p27, antigenic site ø, and α2α3β3β4(AM14) among the RSV/B isolates. The distribution of entropy values among the amino acids in the F protein of RSV/A and RSV/B was similar (Fig 3). For both RSV/A and RSV/B, most of the entropy values in the amino acids of antigenic sites were low, at ≤0.1. Higher entropy values (>0.1) were observed more frequently in RSV/A (N = 27) than RSV/B (N = 19) viruses.

**Table 10:** Amino acids of the RSV fusion gene with the greatest 5% entropy among isolates in RSV/A and RSV/B subgroups.

RSV/A			RSV/B		
Domain	AA Position	Entropy	Domain	AA Position	Entropy
Transmembrane	540	1.03	Transmembrane	529	0.92
Not Defined 2	105	0.67	Not Defined 2	103	0.58
*Antigenic Site II*	276	0.67	*MPE8*	45	0.53
Heptad Repeat 2	518	0.51	Cytoplasmic Tail	573	0.41
*p27*	124	0.46	Not Defined 4	312	0.4
*p27*	125	0.45	*Antigenic Site ø*	209	0.37
Signal Peptide	16	0.44	Signal Peptide	4	0.34
Transmembrane	547	0.44	Not Defined 4	278	0.31
*p27*	129	0.41	Signal Peptide	7	0.3
Signal Peptide	8	0.4	Heptad Repeat 2	518	0.3
Signal Peptide	20	0.37	Cytoplasmic Tail	551	0.21
Signal Peptide	6	0.34	Signal Peptide	11	0.2
*p27*	122	0.34	Signal Peptide	12	0.2
*Antigenic Site I*	384	0.31	Transmembrane	472	0.2
Signal Peptide	19	0.27	Signal Peptide	8	0.16
Not Defined 4	356	0.27	Signal Peptide	15	0.13
*α2α3β3β4 (AM14)*	169	0.24	Transmembrane	527	0.12
Not Defined 2	101	0.21	*p27*	117	0.11
Not Defined 2	103	0.19	Cytoplasmic Tail	562	0.11
Cytoplasmic Tail	574	0.19	*Antigenic Site ø*	65	0.1
Signal Peptide	2	0.18	*p27*	114	0.1
Signal Peptide	13	0.18	*α2α3β3β4 (AM14)*	172	0.1
Signal Peptide	15	0.17	Cytoplasmic Tail	564	0.1
Not Defined 4	377	0.17	*p27*	125	0.09
Signal Peptide	4	0.13	Transmembrane	526	0.09
*p27*	117	0.13	Signal Peptide	16	0.08
Signal Peptide	22	0.11	Signal Peptide	22	0.08
*α2α3β3β4 (AM14)*	152	0.1	Not Defined 1	39	0.08
Not Defined 3	213	0.1	*α2α3β3β4 (AM14)*	178	0.08
Transmembrane	535	0.1	Not Defined 4	291	0.08

Entropy was defined as ∑_*i*_−*i* log(*i*). Individual genotypes of each subgroup contributed equally to the proportion of amino acids found at a given residue.

## Discussion

The F protein of RSV is the central antigen for RSV vaccine development. Because most vaccines in development are monovalent and based on a historical sequences of the GA1 genotype of RSV/A, we chose the historical RSV/A *Long* strain of RSV/A as our reference sequence, which was orginially isolated in 1956. We utilized 1,090 sequences from GenBank that were obtained over the past 6 decades from various locations throughout the world and took several steps to ensure our sequences were assigned the correct genotype (S1 Fig). Focus was given to the analysis of amino acids, opposed to nucleotide, due to the relatvely high rate or variability as well as functionality of the molecules. Examining the antigenic sites of RSV/A and RSV/B sequences to the RSV/A *Long* strain we found that, while these sites are generally well conserved, differences did exist and were most pronounced among the pre-fusion sites of RSV/B. The amino acid changes observed in the antigenic sites occurred at a frequency of 90% or higher in the RSV/B sequences, and fewer changes were detected in RSV/A sequences. This may indicate that a monovalent RSV F vaccine that is based on the historical GA1 genotype or a contemporary RSV/A genotype may provide a lower efficacy against infections caused by viruses from RSV/B compared to viruses from RSV/A.

In adults who have been infected multiple times with RSV during their life time, the majority of neutralizing antibodies against RSV that are found in sera target the pre-fusion F [26]. Monoclonal antibodies directed at site ø, which is unique to pre-fusion F, have greater neutralization capacity than palivizumab, which is directed against site II. Site II is found in both the pre-fusion and post-fusion forms of F. Palivizumab, a monoclonal antibody that targets site II, is licensed for the prevention of severe RSV infection in high-risk infants born prematurely or have chronic lung disease or hemodynamically significant congenital heart disease. For this reason, both the pre-fusion and post-fusion F are intriguing targets for vaccine development. However, we have found pre-fusion sites to be the most variable of RSV antigenic sites. Indeed, it has been hypothesized that variability in the antigenic site ø of RSV/A and RSV/B may result in subgroup specific immunity. Although subgroup specific epitopes have been identified, the neutralizing potential of monoclonals developed against the prefusion antigenic sites is not well defined [27].

Site p27 was found to be the most variable antigenic site among both RSV/A and RSV/B. The F protein of RSV is unique in that it contains two furin sites that are cleaved to form a fully activated pre-fusion F. p27 is the cleavage product that is removed when furin-like protease cleave at its surrounding two furin sites. Cleavage at the two furin sites of the F protein was thought to occur as a post-transcriptional process, with the fully cleaved F being transported to the cell surface. Recently, it has been described that the second furin site does not undergo cleavage until the virus infects the cell and is internalized by macropinocytosis. After internalization the second furin site is cleaved making the virus fully infectious [28]. If this RSV entry mechanism is correct, it would indicate that an intermidate F that still possess p27 is present on the respiratory epithelial cell surface as the virus is budding and being released into the respiratory secretions. It would also indicate that virions with intermidate F containing the p27 will be exposed to the host immune response during RSV infection. Fuentes et al recently demonstrated p27 to be a dominant antigenic site recognized by sera from children and adults infected with RSV [16]. Among young children, there was significantly greater binding acitivty in sera for the p27 epitope than other antigenic sites, including site II and site IV [16]. Its great variability between RSV/A and RSV/B may hint at subgroup specific immunity.

Evaluating the entropy of RSV/A and RSV/B allows us to understand the variability of sequences within each subgroup and also to compare the two. When examining the amino acids within the top 5% of entropy, we found RSV/A to be more variable than RSV/B and to have a greater number of residues with higher entropy values (>0.1). This is consistant with studies of overall variability in the F gene of the two subgroups [29,30]. Both RSV/A and RSV/B had high entropy value amino acids in the same non-antigenic site domains. However, they differed in the high entropy value amino acids of antigenic sites. Both subgroups had amino acids with high entropy values in p27 and α2α3β3β4(AM14). However, among RSV/A isolates, amino acids with high entropy values were found in antigenic sites I and II and among RSV/B isolates, amino acids with high entropy values were found in antigenic sites ø and MPE-8. Some antigenic sites may be more conserved within each subgroup, if not subgroup specific.

Our study is limited in the type and number of sequences available to us; however, this is the largest collection to-date that analyses the sequences of the F gene. Our data also skews towards more recent collection years, as the cost of sequencing has become more inexpensive during this time. For this reason, some genotypes are more represented than others. To help balance over representation and underrepresentation of viruses in different genotypes in our subgroup analysis equal weight was given to each genotype. Likewise, the number of RSV/A sequences reported in this study is approximately three times that of RSV/B. This might be a selection bias based on the sequences in GenBank or it might represent the seasonal variability by location with RSV/A isolates being the predominate viruses. Most of the antigenic site changes observed among the genotypes of RSV/A and RSV/B are conserved; although some genotypes appear to be more susceptible to change. This might be attributed to the smaller sample sizes of some of these genotypes. Additionally, the occurence of low-level variability appears more frequently among the genotypes of RSV/A. This could be due to the overall larger size of the RSV/A subgroup. In addition, we are limited in that the corresponding G genes were not available for all of our F genes. A further limitation was the quality of sequencing available for our consumption. While we believe our system of genotyping based on only the F gene to be sufficient, some misclassification is possible.

In summary, this study documents that most of the known antigenic sites of RSV F are generally well conserved; though, differences do exist when comparing the two subgroups to the reference RSV/A *Long* strain. Additionally, we found a number of differences in non-antigenic sites. Perhaps, containing some antigenic domains yet to be identified. To our surprise, the non-synmoynous changes in the antigenic sites that were detected in the RSV/B isolates occurred at nearly 100% frequency. The signficance of these non-synmoynous changes in the antigenic domains is unclear. Ongoing RSV F vaccine trials primarily with monovalent formulation will provide insight on the importance of these observed differences, and could impact the next generation of RSV F vaccine formulation.

## Supporting information

S1 Table Accession numbers for sequences acquired from GenBank. (DOCX)

S2 Table Frequency of amino acid changes in non-antigenic domains of the fusion gene for RSV/A (N = 822) and RSV/B (N = 268) compared to the RSV/A *Long* strain. (DOCX)

S3 Table Frequency of nucleotide changes in antigenic sites of the fusion gene for RSV/A (N = 822) and RSV/B (N = 268) compared to the RSV/A *Long* strain. (DOCX)

S1 Fig RSV fusion gene sequence collection strategy. (TIF)

S2 Fig Phylogenetic trees for the attachment protein of RSV/A. (TIF)

S3 Fig Phylogenetic trees for the attachment protein of RSV/B. (TIF)

S4 Fig Phylogenetic trees for the fusion protein of RSV/A. (TIF)

S5 Fig Phylogenetic trees for the fusion protein of RSV/B. (TIF)
