# Correction: Sequence variability of the respiratory syncytial virus (RSV) fusion gene among contemporary and historical genotypes of RSV/A and RSV/B

**DOI:** 10.1371/journal.pone.0180623

**Published:** 2017-06-28

**Authors:** Anne M. Hause, David M. Henke, Vasanthi Avadhanula, Chad A. Shaw, Lorena I. Tapia, Pedro A. Piedra

Fig 3 appears incorrectly in the published article. Please see the correct Fig 3 here.

**Fig 3:**
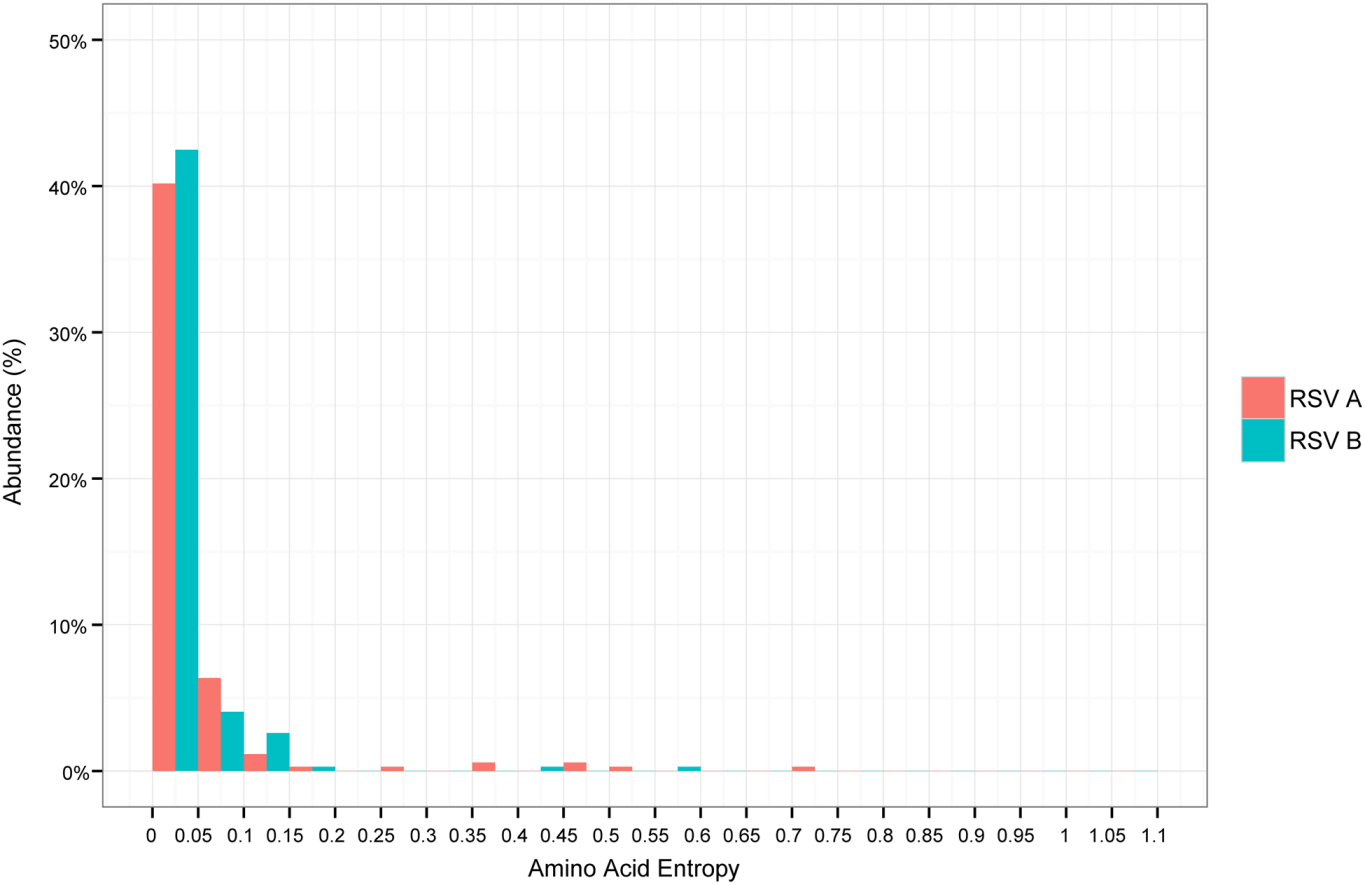
The entropy values in amino acids within antigenic sites of the fusion gene of RSV/A and RSV/B have a similar distribution. Entropy was defined as ∑_*i*_−*i* log(*i*). Individual genotypes of each subgroup contributed equally to the proportion of amino acids found at a given residue.
