# Supplementary material for: Sequence variability of the respiratory syncytial virus (RSV) fusion gene among contemporary and historical genotypes of RSV/A and RSV/B
Source: PLoS One. 2017 Apr 17;12(4):e0175792. doi: 10.1371/journal.pone.0175792 (PMC5393888; doi:10.1371/journal.pone.0175792)
Supplement: S1 Table — (DOCX) [file pone.0175792.s001.docx]

**S1 Table: Accession numbers for sequences acquired from GenBank**

| **GenBank Accession ID** |  | **GenBank Accession ID** |  | **GenBank Accession ID** |  | **GenBank Accession ID** |  | **GenBank Accession ID** |  | **GenBank Accession ID** |
| --- | --- | --- | --- | --- | --- | --- | --- | --- | --- | --- |
| AY198175 |  | KJ627287 |  | KJ643463 |  | KM042383 |  | JX682778 |  | JX576750 |
| AY198176 |  | KJ627288 |  | KJ643464 |  | KM042384 |  | JX682779 |  | JX576751 |
| AY198177 |  | KJ627289 |  | KJ643465 |  | KM042385 |  | JX682780 |  | JX576752 |
| AY911262 |  | KJ627290 |  | KJ643466 |  | KM042386 |  | JX682781 |  | JX576753 |
| GU591758 |  | KJ627291 |  | KJ643467 |  | KM042388 |  | JX682782 |  | JX576754 |
| GU591759 |  | KJ627292 |  | KJ643468 |  | KM042389 |  | JX682783 |  | JX576755 |
| GU591760 |  | KJ627293 |  | KJ643469 |  | KM042390 |  | JX682784 |  | JX576756 |
| GU591761 |  | KJ627294 |  | KJ643470 |  | KM042391 |  | JX682785 |  | JX576757 |
| GU591762 |  | KJ627295 |  | KJ643471 |  | KM042392 |  | JX682786 |  | JX576758 |
| GU591763 |  | KJ627296 |  | KJ643472 |  | KM042393 |  | JX682787 |  | JX576759 |
| GU591764 |  | KJ627297 |  | KJ643473 |  | KM517572 |  | JX682788 |  | JX576760 |
| GU591765 |  | KJ627298 |  | KJ643474 |  | KM517573 |  | JX682789 |  | JX576761 |
| GU591766 |  | KJ627299 |  | KJ643475 |  | KM578843 |  | JX682790 |  | JX576762 |
| GU591767 |  | KJ627300 |  | KJ643476 |  | KP218910 |  | JX682791 |  | JX682715 |
| GU591768 |  | KJ627301 |  | KJ643477 |  | KP258695 |  | JX682792 |  | JX682716 |
| GU591769 |  | KJ627302 |  | KJ643478 |  | KP258696 |  | JX682793 |  | JX682717 |
| GU591770 |  | KJ627303 |  | KJ643479 |  | KP258697 |  | JX682794 |  | JX682718 |
| GU591771 |  | KJ627304 |  | KJ643480 |  | KP258698 |  | JX682795 |  | JX682719 |
| JF421562 |  | KJ627305 |  | KJ643481 |  | KP258700 |  | JX682796 |  | JX682720 |
| JF714709 |  | KJ627306 |  | KJ643482 |  | KP258701 |  | JX682797 |  | JX682721 |
| JF714710 |  | KJ627307 |  | KJ643483 |  | KP258702 |  | JX682798 |  | JX682722 |
| JF714712 |  | KJ627308 |  | KJ643484 |  | KP258703 |  | JX682799 |  | JX682723 |
| JF920055 |  | KJ627309 |  | KJ643485 |  | KP258704 |  | JX682800 |  | JX682724 |
| JF920056 |  | KJ627310 |  | KJ643486 |  | KP258705 |  | JX682801 |  | JX682725 |
| JF920063 |  | KJ627311 |  | KJ643487 |  | KP258707 |  | JX682802 |  | JX682726 |
| JF920066 |  | KJ627312 |  | KJ643488 |  | KP258708 |  | JX682803 |  | JX682727 |
| JF920067 |  | KJ627313 |  | KJ643489 |  | KP258709 |  | JX682804 |  | JX682728 |
| JF920068 |  | KJ627314 |  | KJ643490 |  | KP258710 |  | JX682805 |  | JX682729 |
| JF920070 |  | KJ627315 |  | KJ643491 |  | KP258711 |  | JX682806 |  | JX682730 |
| JN032115 |  | KJ627316 |  | KJ643492 |  | KP258712 |  | JX682807 |  | JX682731 |
| JN032116 |  | KJ627317 |  | KJ643493 |  | KP258713 |  | JX682808 |  | JX682732 |
| JN032117 |  | KJ627318 |  | KJ643494 |  | KP258714 |  | JX682809 |  | JX682733 |
| JN032118 |  | KJ627319 |  | KJ643495 |  | KP258715 |  | JX682810 |  | JX682734 |
| JN032119 |  | KJ627320 |  | KJ643496 |  | KP258717 |  | JX682811 |  | JX682735 |
| JN032120 |  | KJ627321 |  | KJ643497 |  | KP258718 |  | JX682812 |  | JX682736 |
| JN032122 |  | KJ627322 |  | KJ643498 |  | KP258719 |  | JX682813 |  | JX682737 |
| JQ582843 |  | KJ627324 |  | KJ643499 |  | KP258720 |  | JX682814 |  | JX682738 |
| JQ582844 |  | KJ627325 |  | KJ643500 |  | KP258721 |  | JX682815 |  | JX682739 |
| JX576729 |  | KJ627326 |  | KJ643501 |  | KP258722 |  | JX682816 |  | JX682740 |
| JX576730 |  | KJ627327 |  | KJ643502 |  | KP258723 |  | JX682817 |  | JX682741 |
| JX576731 |  | KJ627328 |  | KJ643503 |  | KP258724 |  | JX682818 |  | JX682742 |
| JX576732 |  | KJ627329 |  | KJ643504 |  | KP258725 |  | JX682819 |  | JX682743 |
| JX576733 |  | KJ627330 |  | KJ643505 |  | KP258726 |  | JX682820 |  | JX682744 |
| JX576734 |  | KJ627331 |  | KJ643506 |  | KP258727 |  | JX682821 |  | JX682745 |
| JX576735 |  | KJ627332 |  | KJ643507 |  | KP258728 |  | JX682822 |  | JX682746 |
| JX576736 |  | KJ627333 |  | KJ643508 |  | KP258729 |  | JX682823 |  | JX682747 |
| JX576737 |  | KJ627334 |  | KJ643510 |  | KP258730 |  | JF503241 |  | JX682748 |
| JX576738 |  | KJ627335 |  | KJ643511 |  | KP258731 |  | JF503242 |  | JX682749 |
| JX576739 |  | KJ627336 |  | KJ643512 |  | KP258732 |  | JF421560 |  | JX682750 |
| JX576740 |  | KJ627337 |  | KJ643513 |  | KP258733 |  | JF421561 |  | JX682751 |
| JX576741 |  | KJ627338 |  | KJ643514 |  | KP258734 |  | JF421563 |  | JX682752 |
| JX576742 |  | KJ627339 |  | KJ643515 |  | KP258735 |  | JF421565 |  | JX682753 |
| JX576743 |  | KJ627340 |  | KJ643517 |  | KP258736 |  | JQ736673 |  | JX682754 |
| JX576744 |  | KJ627341 |  | KJ643518 |  | KP258737 |  | JQ736674 |  | KC618407 |
| JX576745 |  | KJ627342 |  | KJ643519 |  | KP258738 |  | JQ736675 |  | KC618408 |
| JX576746 |  | KJ627343 |  | KJ643520 |  | KP258739 |  | JQ736676 |  | KC618409 |
| KC731482 |  | KJ627344 |  | KJ643521 |  | KP258740 |  | JQ736677 |  | KC618410 |
| KC731483 |  | KJ627345 |  | KJ643522 |  | KP258741 |  | JQ736678 |  | KF246616 |
| KC978856 |  | KJ627346 |  | KJ643523 |  | KP258742 |  | JQ736679 |  | KF530260 |
| KF246594 |  | KJ627348 |  | KJ643524 |  | KP258743 |  | JQ736680 |  | KF530261 |
| KF246602 |  | KJ627349 |  | KJ643525 |  | KP258744 |  | KF246597 |  | KF530268 |
| KF246605 |  | KJ627350 |  | KJ643526 |  | KP258745 |  | KF246603 |  | KF530269 |
| KF246607 |  | KJ627351 |  | KJ643527 |  | KP317916 |  | KF246628 |  | KF826816 |
| KF246608 |  | KJ627352 |  | KJ643528 |  | KP317917 |  | KF246635 |  | KF826817 |
| KF246611 |  | KJ627353 |  | KJ643529 |  | KP317919 |  | KF246609 |  | KF826821 |
| KF246613 |  | KJ627354 |  | KJ643530 |  | KP317920 |  | JX171068 |  | KF826823 |
| KF246614 |  | KJ627355 |  | KJ643531 |  | KP317921 |  | JX171069 |  | KF826824 |
| KF246615 |  | KJ627356 |  | KJ643532 |  | KP317922 |  | JX171070 |  | KF826826 |
| KF246617 |  | KJ627357 |  | KJ643533 |  | KP317923 |  | JX171071 |  | KF826827 |
| KF246618 |  | KJ627358 |  | KJ643534 |  | KP317925 |  | JX171072 |  | KF826828 |
| KF246619 |  | KJ627359 |  | KJ643535 |  | KP317926 |  | JX171073 |  | KF826830 |
| KF246621 |  | KJ627360 |  | KJ643536 |  | KP317927 |  | JN257682 |  | KF826831 |
| KF246623 |  | KJ627361 |  | KJ643537 |  | KP317928 |  | JN257683 |  | KF826832 |
| KF246624 |  | KJ627362 |  | KJ643538 |  | KP317929 |  | JN257684 |  | KF826833 |
| KF246626 |  | KJ627364 |  | KJ643540 |  | KP317930 |  | JN257685 |  | KF826836 |
| KF246627 |  | KJ627365 |  | KJ643541 |  | KP317932 |  | JN257686 |  | KF826837 |
| KF246630 |  | KJ627366 |  | KJ643542 |  | KP317933 |  | JN257687 |  | KF826838 |
| KF246632 |  | KJ627367 |  | KJ643543 |  | KP317934 |  | JN257688 |  | KF826840 |
| KF246636 |  | KJ627369 |  | KJ643544 |  | KP317935 |  | JN257689 |  | KF826841 |
| KF246643 |  | KJ627370 |  | KJ643545 |  | KP317936 |  | JN257690 |  | KF826846 |
| KF246644 |  | KJ627371 |  | KJ643546 |  | KP317937 |  | JN257691 |  | KF826847 |
| KF530259 |  | KJ627372 |  | KJ643547 |  | KP317938 |  | JN257692 |  | KF826848 |
| KF530262 |  | KJ627373 |  | KJ643548 |  | KP317939 |  | AB848366 |  | KF826849 |
| KF530266 |  | KJ627374 |  | KJ643549 |  | KP317941 |  | AB848367 |  | KF826850 |
| KF826819 |  | KJ627647 |  | KJ643550 |  | KP317942 |  | AB848368 |  | KF826852 |
| KF826820 |  | KJ627648 |  | KJ643551 |  | KP317944 |  | AB848369 |  | KF826854 |
| KF826822 |  | KJ627649 |  | KJ643552 |  | KP317945 |  | AB848370 |  | KF826855 |
| KF826825 |  | KJ627652 |  | KJ643553 |  | KP317946 |  | AB848371 |  | KF826856 |
| KF826829 |  | KJ627653 |  | KJ643554 |  | KP317949 |  | AB848372 |  | KF973331 |
| KF826834 |  | KJ627654 |  | KJ643555 |  | KP317950 |  | AB848373 |  | KJ130649 |
| KF826835 |  | KJ627656 |  | KJ643556 |  | KP317951 |  | AB848374 |  | KJ130650 |
| KF826839 |  | KJ627657 |  | KJ643557 |  | KP317952 |  | AY526558 |  | KJ130651 |
| KF826842 |  | KJ627658 |  | KJ643558 |  | KP317953 |  | AY526559 |  | KJ130652 |
| KF826843 |  | KJ627659 |  | KJ643559 |  | KP317954 |  | AY526560 |  | KJ130653 |
| KF826844 |  | KJ627662 |  | KJ643560 |  | KP317955 |  | AY526561 |  | KJ130654 |
| KF826845 |  | KJ627663 |  | KJ643561 |  | KP317956 |  | AY526562 |  | KJ672424 |
| KF826851 |  | KJ627664 |  | KJ643562 |  | KP663729 |  | AY526563 |  | KJ672426 |
| KF826853 |  | KJ627666 |  | KJ643563 |  | KP663730 |  | AY526564 |  | KJ672427 |
| KF826857 |  | KJ627667 |  | KJ643564 |  | KP856961 |  | AY526565 |  | KJ672428 |
| KF826858 |  | KJ627668 |  | KJ643565 |  | KP856963 |  | AY526566 |  | KJ672429 |
| KF826859 |  | KJ627669 |  | KJ643566 |  | KP856965 |  | AY526567 |  | KJ672431 |
| KF826860 |  | KJ627670 |  | KJ643567 |  | KP856966 |  | JF503243 |  | KJ672432 |
| KF973319 |  | KJ627671 |  | KJ643568 |  | KP856967 |  | JF503244 |  | KJ672433 |
| KF973320 |  | KJ627672 |  | KJ643569 |  | KP856968 |  | AF512538 |  | KJ672434 |
| KF973321 |  | KJ627673 |  | KJ643570 |  | KP856969 |  | AY114149 |  | KJ672435 |
| KF973322 |  | KJ627674 |  | KJ643571 |  | KT285064 |  | AY114150 |  | KJ672436 |
| KF973323 |  | KJ627675 |  | KJ643572 |  | JX198138 |  | AY114151 |  | KJ672437 |
| KF973324 |  | KJ627676 |  | KJ643573 |  | JX627336 |  | AY526556 |  | KJ672439 |
| KF973325 |  | KJ627678 |  | KJ643574 |  | KP663728 |  | AY526557 |  | KJ672440 |
| KF973326 |  | KJ627679 |  | KJ643575 |  | D00151 |  | JF920046 |  | KJ672441 |
| KF973327 |  | KJ627680 |  | KJ643576 |  | JX171066 |  | JF920047 |  | KJ672442 |
| KF973328 |  | KJ627681 |  | KJ643577 |  | JX171067 |  | JF920048 |  | KJ672443 |
| KF973329 |  | KJ627682 |  | KJ643578 |  | AY526550 |  | JF920049 |  | KJ672444 |
| KF973330 |  | KJ627683 |  | KJ643579 |  | AY526551 |  | JF920050 |  | KJ672446 |
| KF973332 |  | KJ627684 |  | KJ643580 |  | AY526552 |  | JF920051 |  | KJ672447 |
| KF973333 |  | KJ627685 |  | KJ643581 |  | AY526553 |  | JF920052 |  | KJ672448 |
| KF973334 |  | KJ627686 |  | KJ643583 |  | AY526554 |  | JF920053 |  | KJ672449 |
| KF973335 |  | KJ627687 |  | KJ643584 |  | AY526555 |  | JF920054 |  | KJ672450 |
| KF973336 |  | KJ627688 |  | KJ643585 |  | DQ885231 |  | JF920057 |  | KJ672451 |
| KF973338 |  | KJ627689 |  | KJ643586 |  | JX482018 |  | JF920058 |  | KJ672452 |
| KF973339 |  | KJ627690 |  | KJ643587 |  | JX482019 |  | JF920062 |  | KJ672453 |
| KF973340 |  | KJ627693 |  | KJ643588 |  | JX482020 |  | JF920065 |  | KJ672454 |
| KJ627245 |  | KJ627695 |  | KJ643589 |  | JX482021 |  | JF920069 |  | KJ672455 |
| KJ627246 |  | KJ627696 |  | KJ672425 |  | JX482022 |  | JQ901447 |  | KJ672456 |
| KJ627247 |  | KJ627697 |  | KJ672430 |  | JX482023 |  | JQ901448 |  | KJ672457 |
| KJ627248 |  | KJ627698 |  | KJ672438 |  | JX482024 |  | JQ901449 |  | KJ672458 |
| KJ627249 |  | KJ627699 |  | KJ672473 |  | JX482025 |  | JQ901450 |  | KJ672459 |
| KJ627250 |  | KJ627701 |  | KJ672476 |  | JX482026 |  | JQ901451 |  | KJ672460 |
| KJ627251 |  | KJ627702 |  | KJ672481 |  | JX482027 |  | JQ901452 |  | KJ672461 |
| KJ627252 |  | KJ627703 |  | KJ723460 |  | JX482028 |  | JQ901453 |  | KJ672462 |
| KJ627253 |  | KJ627704 |  | KJ723461 |  | JX482029 |  | JQ901454 |  | KJ672463 |
| KJ627254 |  | KJ627705 |  | KJ723462 |  | JX482030 |  | JQ901455 |  | KJ672464 |
| KJ627255 |  | KJ627706 |  | KJ723463 |  | JX482031 |  | JQ901456 |  | KJ672465 |
| KJ627256 |  | KJ627707 |  | KJ723464 |  | JX482032 |  | JQ901457 |  | KJ672466 |
| KJ627257 |  | KJ627708 |  | KJ723465 |  | JX482033 |  | JQ901458 |  | KJ672467 |
| KJ627258 |  | KJ627709 |  | KJ723466 |  | JX482034 |  | JX015479 |  | KJ672468 |
| KJ627259 |  | KJ627710 |  | KJ723467 |  | JX482035 |  | JX015480 |  | KJ672469 |
| KJ627260 |  | KJ627711 |  | KJ723468 |  | JX482036 |  | JX015481 |  | KJ672470 |
| KJ627261 |  | KJ627714 |  | KJ723469 |  | JX482037 |  | JX015483 |  | KJ672471 |
| KJ627262 |  | KJ627715 |  | KJ723470 |  | JX482038 |  | JX015484 |  | KJ672472 |
| KJ627263 |  | KJ627716 |  | KJ723472 |  | JX682755 |  | JX015485 |  | KJ672474 |
| KJ627264 |  | KJ627717 |  | KJ723473 |  | JX682756 |  | JX015486 |  | KJ672475 |
| KJ627266 |  | KJ627718 |  | KJ723474 |  | JX682757 |  | JX015487 |  | KJ672477 |
| KJ627267 |  | KJ627719 |  | KJ723475 |  | JX682758 |  | JX015488 |  | KJ672478 |
| KJ627268 |  | KJ627720 |  | KJ723476 |  | JX682759 |  | JX015489 |  | KJ672479 |
| KJ627269 |  | KJ627721 |  | KJ723477 |  | JX682760 |  | JX015490 |  | KJ672480 |
| KJ627270 |  | KJ627722 |  | KJ723478 |  | JX682761 |  | JX015492 |  | KJ672482 |
| KJ627271 |  | KJ627723 |  | KJ723479 |  | JX682762 |  | JX015493 |  | KJ672483 |
| KJ627272 |  | KJ627724 |  | KJ723480 |  | JX682763 |  | JX015494 |  | KJ672484 |
| KJ627273 |  | KJ627725 |  | KJ723481 |  | JX682764 |  | JX015495 |  | KP258699 |
| KJ627274 |  | KJ627727 |  | KJ723482 |  | JX682765 |  | JX015496 |  | M22643 |
| KJ627275 |  | KJ627728 |  | KJ723483 |  | JX682766 |  | JX015497 |  | U31558 |
| KJ627276 |  | KJ627729 |  | KJ723484 |  | JX682767 |  | JX015498 |  | U31559 |
| KJ627277 |  | KJ627731 |  | KJ723485 |  | JX682768 |  | JX015499 |  | U31560 |
| KJ627278 |  | KJ627732 |  | KJ723486 |  | JX682769 |  | JX069798 |  | U31561 |
| KJ627279 |  | KJ627733 |  | KJ723487 |  | JX682770 |  | JX069799 |  | U31562 |
| KJ627280 |  | KJ627734 |  | KJ723488 |  | JX682771 |  | JX069800 |  | JX198136 |
| KJ627281 |  | KJ627735 |  | KJ723489 |  | JX682772 |  | JX069801 |  | JX198113 |
| KJ627282 |  | KJ627736 |  | KJ723490 |  | JX682773 |  | JX069802 |  |  |
| KJ627283 |  | KJ627737 |  | KJ723491 |  | JX682774 |  | JX069803 |  |  |
| KJ627284 |  | KJ627738 |  | KJ723492 |  | JX682775 |  | JX576747 |  |  |
| KJ627285 |  | KJ641590 |  | KM042381 |  | JX682776 |  | JX576748 |  |  |
| KJ627286 |  | KJ643462 |  | KM042382 |  | JX682777 |  | JX576749 |  |  |
