# Supplementary material for: Sequence variability of the respiratory syncytial virus (RSV) fusion gene among contemporary and historical genotypes of RSV/A and RSV/B
Source: PLoS One. 2017 Apr 17;12(4):e0175792. doi: 10.1371/journal.pone.0175792 (PMC5393888; doi:10.1371/journal.pone.0175792)
Supplement: S2 Table — (DOCX) [file pone.0175792.s002.docx]

**S2 Table: Frequency of amino acid changes in non-antigenic domains for RSV/A (N= 822) and RSV/B (N=268) compared to the RSV/A *Long* strain.**

| Fusion Domain Name | Amino Acids | RSV/A | | RSV/B | |
| --- | --- | --- | --- | --- | --- |
|  |  | **AA Change** | **Frequency** | **AA Change** | **Frequency** |
| Signal Peptide | 1-25 | - | - | P4L | 92% |
|  |  | - | - | P4M | 7% |
|  |  | L6I | 7% | L6H | 99% |
|  |  | - | - | K7R | 92% |
|  |  | A8T | 88% | A8S | 96% |
|  |  | - | - | N9S | 100% |
|  |  | - | - | T12F | 95% |
|  |  | - | - | T13L | 100% |
|  |  | - | - | I14T | 100% |
|  |  | A16I | 7% | - | - |
|  |  | - | - | A17I | 100% |
|  |  | - | - | V18N | 100% |
|  |  | F20L | 88% | F20L | 100% |
|  |  | - | - | C21Y | 100% |
|  |  | - | - | F22L | 99% |
|  |  | - | - | A23T | 100% |
|  | | | | | |
| Undefined | 26-52 | - | - | K42R | 100% |
|  | | | | | |
| Heptad Repeat 3 | 53-100 | - | - | A74T | 100% |
|  |  | N80K | 100% | N80K | 100% |
|  |  | - | - | S99N | 100% |
|  | | | | | |
| Undefined | 101-105 | T101P | 96% | T101P | 100% |
|  |  | - | - | A103V | 14% |
|  |  | N105S | 40% | - | - |
|  | | | | | |
| Undefined | 207-253 | R213S | 98% | - | - |
|  |  | - | - | N228S | 100% |
|  |  | - | - | V247L | 100% |
|  | | | | | |
| Undefined | 278-379 | - | - | P312H | 9% |
|  |  | - | - | T326I | 100% |
|  |  | E356D | 8% | E356D | 100% |
|  | | | | | |
| Undefined | 401-421 | - | - | V402I | 99% |
|  | | | | | |
| Undefined | 439-473 | - | - | Q462L | 100% |
|  |  | - | - | S466N | 100% |
|  | | | | | |
| Heptad Repeat 2 | 474-523 | - | - | F477Y | 100% |
|  |  | - | - | K508R | 100% |
|  |  | H515N | 99% | H515N | 100% |
|  |  | A518V | 18% | A518T | 91% |
|  | | | | | |
| Transmembrane | 524-548 | - | - | T529A | 61% |
|  |  | - | - | T529V | 14% |
|  |  | - | - | I537V | 100% |
|  |  | S540A | 38% | - | - |
|  |  | S540L | 17% | - | - |
|  |  | - | - | V544I | 100% |
|  |  | L547F | 16% | - | - |
|  | | | | | |
| Cytoplasmic Tail | 549-574 | - | - | R553K | 100% |
|  |  | - | - | S554N | 99% |
|  |  | - | - | S573R | 14% |
|  |  | - | - | N574K | 100% |

Changes with ≤5% frequency were omitted from this table. Individual genotypes of each subgroup contributed equally to the proportion of amino acids found at each residue.
