# Supplementary material for: Sequence variability of the respiratory syncytial virus (RSV) fusion gene among contemporary and historical genotypes of RSV/A and RSV/B
Source: PLoS One. 2017 Apr 17;12(4):e0175792. doi: 10.1371/journal.pone.0175792 (PMC5393888; doi:10.1371/journal.pone.0175792)
Supplement: S3 Table — (DOCX) [file pone.0175792.s003.docx]

**S3 Table: Frequency of nucleotide changes in antigenic sites of the fusion gene for RSV/A (N= 822) and RSV/B (N=268) compared to the RSV/A *Long* strain.**

| Antigenic Site | Nucleotides | RSV/A | | RSV/B | |
| --- | --- | --- | --- | --- | --- |
|  |  | **Ntd Change** | **Frequency** | **Ntd Change** | **Frequency** |
| **I** | 1138-1200 | - | - | A1138C | 14% |
|  |  | - | - | A1139G | 85% |
|  |  | - | - | T1140C | 84% |
|  |  | - | - | T1140G | 14% |
|  |  | - | - | T1142C | 14% |
|  |  | - | - | C1143T | 98% |
|  |  | - | - | G1145T | 14% |
|  |  | - | - | C1146T | 85% |
|  |  | - | - | C1146G | 15% |
|  |  | - | - | A1147T | 15% |
|  |  | T1149C | 85% | T1149C | 85% |
|  |  | - | - | T1149A | 15% |
|  |  | G1150A | 88% | G1150A | 85% |
|  |  | - | - | G1150C | 14% |
|  |  | - | - | T1151C | 86% |
|  |  | - | - | T1151A | 14% |
|  |  | T1152A | 7% | T1152C | 16% |
|  |  | - | - | G1153T | 15% |
|  |  | - | - | A1154G | 14% |
|  |  | - | - | C1155A | 14% |
|  |  | - | - | A1156C | 14% |
|  |  | - | - | T1157A | 14% |
|  |  | - | - | A1158T | 14% |
|  |  | - | - | T1159A | 15% |
|  |  | - | - | C1161T | 14% |
|  |  | - | - | A1162C | 15% |
|  |  | T1164C | 87% | T1164A | 14% |
|  |  | - | - | C1165T | 98% |
|  |  | - | - | C1166T | 14% |
|  |  | C1167T | 24% | - | - |
|  |  | - | - | A1168C | 14% |
|  |  | - | - | A1170G | 82% |
|  |  | - | - | T1171G | 14% |
|  |  | - | - | A1172T | 14% |
|  |  | - | - | T1173A | 15% |
|  |  | - | - | T1173C | 6% |
|  |  | - | - | G1174T | 14% |
|  |  | - | - | A1175G | 14% |
|  |  | - | - | T1176C | 83% |
|  |  | - | - | T1176A | 14% |
|  |  | - | - | T1177C | 15% |
|  |  | - | - | G1178T | 14% |
|  |  | T1179C | 86% | T1179C | 85% |
|  |  | - | - | T1179G | 14% |
|  |  | - | - | A1180C | 14% |
|  |  | - | - | T1184A | 14% |
|  |  | - | - | A1186T | 14% |
|  |  | - | - | T1187A | 14% |
|  |  | - | - | G1188T | 15% |
|  |  | - | - | A1189G | 15% |
|  |  | - | - | C1190A | 14% |
|  |  | - | - | T1191A | 85% |
|  |  | - | - | T1191C | 14% |
|  |  | - | - | T1192A | 15% |
|  |  | - | - | C1193T | 14% |
|  |  | - | - | A1194C | 14% |
|  |  | - | - | C1199A | 14% |
|  |  | - | - | A1200C | 14% |
|  | | | | | |
| **II** | 760-831 | - | - | T762C | 85% |
|  |  | - | - | T762A | 15% |
|  |  | - | - | A763C | 14% |
|  |  | - | - | G764A | 14% |
|  |  | - | - | T765G | 14% |
|  |  | - | - | G766T | 15% |
|  |  | - | - | A767G | 14% |
|  |  | A768G | 40% | A768G | 85% |
|  |  | - | - | T769G | 12% |
|  |  | - | - | A771T | 14% |
|  |  | - | - | T772C | 85% |
|  |  | - | - | T772A | 15% |
|  |  | - | - | T773C | 15% |
|  |  | G774A | 86% | G774A | 86% |
|  |  | - | - | T775A | 15% |
|  |  | - | - | C776T | 15% |
|  |  | - | - | A777C | 14% |
|  |  | - | - | T778A | 15% |
|  |  | - | - | A780G | 57% |
|  |  | - | - | A780T | 15% |
|  |  | - | - | A781G | 14% |
|  |  | - | - | T782A | 15% |
|  |  | - | - | C783T | 15% |
|  |  | - | - | A784C | 14% |
|  |  | - | - | T786A | 14% |
|  |  |  |  | G787T | 14% |
|  |  | - | - | A788G | 14% |
|  |  | - | - | T789A | 14% |
|  |  | - | - | T789C | 5% |
|  |  | - | - | A790T | 15% |
|  |  | - | - | T791A | 14% |
|  |  | - | - | G792T | 14% |
|  |  | - | - | C793G | 14% |
|  |  | - | - | T795C | 14% |
|  |  | - | - | A796T | 14% |
|  |  | - | - | T797A | 14% |
|  |  | - | - | A798T | 14% |
|  |  | - | - | C800A | 14% |
|  |  | - | - | A801C | 14% |
|  |  | - | - | T804A | 14% |
|  |  | - | - | G805T | 14% |
|  |  | - | - | A806G | 14% |
|  |  | - | - | T807A | 15% |
|  |  | - | - | T807C | 6% |
|  |  | - | - | C808T | 15% |
|  |  | - | - | A809C | 14% |
|  |  | - | - | G810A | 15% |
|  |  | - | - | A811G | 14% |
|  |  | - | - | G816A | 97% |
|  |  | - | - | T817A | 15% |
|  |  | - | - | A819T | 14% |
|  |  | - | - | T821A | 14% |
|  |  | - | - | G822T | 14% |
|  |  | - | - | T823G | 14% |
|  |  | - | - | C824T | 14% |
|  |  | - | - | C825A | 86% |
|  |  | - | - | A827G | 84% |
|  |  | - | - | C828G | 14% |
|  |  | - | - | A829C | 14% |
|  |  | - | - | T831A | 15% |
|  | | | | | |
| **IV** | 1264-1314 | - | - | T1264A | 14% |
|  |  | - | - | G1265T | 15% |
|  |  | - | - | T1266C | 81% |
|  |  | - | - | T1266G | 14% |
|  |  | - | - | A1267C | 14% |
|  |  | - | - | C1268A | 15% |
|  |  | - | - | A1269T | 85% |
|  |  | - | - | A1269C | 14% |
|  |  | - | - | G1270T | 14% |
|  |  | - | - | C1271G | 14% |
|  |  | - | - | A1272C | 14% |
|  |  | - | - | T1273A | 15% |
|  |  | - | - | C1274T | 14% |
|  |  | - | - | A1276C | 14% |
|  |  | - | - | T1278C | 82% |
|  |  | - | - | T1278A | 15% |
|  |  | - | - | A1279C | 15% |
|  |  | - | - | T1284A | 14% |
|  |  | - | - | C1285T | 14% |
|  |  | - | - | G1286C | 15% |
|  |  | - | - | T1287G | 14% |
|  |  | - | - | G1288T | 14% |
|  |  | A1290G | 66% | A1290G | 52% |
|  |  | - | - | T1292A | 15% |
|  |  | - | - | C1293T | 98% |
|  |  | - | - | A1294T | 14% |
|  |  | - | - | T1295A | 14% |
|  |  | - | - | A1296T | 14% |
|  |  | - | - | G1299A | 14% |
|  |  | - | - | A1300G | 15% |
|  |  | - | - | C1301A | 15% |
|  |  | - | - | A1302C | 14% |
|  |  | - | - | T1303A | 14% |
|  |  | T1305C | 38% | - | - |
|  |  | - | - | C1307T | 14% |
|  |  | - | - | T1308C | 14% |
|  |  | - | - | A1309T | 14% |
|  |  | C1311T | 10% | C1311T | 85% |
|  |  | - | - | C1311A | 15% |
|  |  | - | - | G1312T | 14% |
|  |  | - | - | G1314T | 85% |
|  | | | | | |
| **p27** | 325-408 | - | - | G326A | 14% |
|  |  | - | - | A327G | 14% |
|  |  | - | - | G328A | 14% |
|  |  | - | - | A329G | 15% |
|  |  | - | - | C331G | 85% |
|  |  | - | - | C331A | 13% |
|  |  | - | - | T332C | 86% |
|  |  | A333G | 13% | A333C | 14% |
|  |  | - | - | C334A | 14% |
|  |  | - | - | A336C | 14% |
|  |  | - | - | A337A | 14% |
|  |  | - | - | G338A | 86% |
|  |  | - | - | G338C | 14% |
|  |  | G339A | 44% | G339A | 22% |
|  |  | - | - | T440G | 13% |
|  |  | - | - | T341A | 86% |
|  |  | - | - | T342A | 14% |
|  |  | - | - | T342C | 6% |
|  |  | - | - | A343T | 14% |
|  |  | - | - | T344A | 15% |
|  |  | - | - | G345T | 14% |
|  |  | - | - | A346G | 14% |
|  |  | - | - | T348C | 86% |
|  |  | - | - | T348A | 14% |
|  |  | - | - | T349C | 16% |
|  |  | - | - | A350T | 14% |
|  |  | - | - | T351C | 72% |
|  |  | - | - | T351A | 14% |
|  |  | - | - | A352C | 14% |
|  |  | - | - | C353A | 14% |
|  |  | - | - | A354C | 14% |
|  |  | - | - | C355A | 98% |
|  |  | - | - | T356A | 14% |
|  |  | - | - | C357T | 14% |
|  |  | - | - | A358C | 14% |
|  |  | C360T | 5% | C360T | 85% |
|  |  | - | - | C360A | 14% |
|  |  | - | - | A361T | 14% |
|  |  | - | - | A362C | 86% |
|  |  | - | - | T363C | 93% |
|  |  | - | - | T363A | 5% |
|  |  | A364G | 9% | A364C | 15% |
|  |  | - | - | C365A | 15% |
|  |  | - | - | C366T | 81% |
|  |  | - | - | A367T | 15% |
|  |  | A372T | 45% | - | - |
|  |  | A372C | 43% | A372C | 84% |
|  |  | - | - | A373C | 100% |
|  |  | C374A | 17% | C374T | 83% |
|  |  | - | - | C375A | 86% |
|  |  | - | - | T378A | 15% |
|  |  | - | - | G379T | 14% |
|  |  | - | - | T380G | 14% |
|  |  | - | - | A381T | 14% |
|  |  | - | - | A382T | 85% |
|  |  | - | - | C383T | 14% |
|  |  | - | - | A384C | 15% |
|  |  | T385G | 13% | T385A | 98% |
|  |  | - | - | T386A | 15% |
|  |  | - | - | A387T | 14% |
|  |  | - | - | G389A | 14% |
|  |  | C390T | 26% | C390G | 15% |
|  |  | - | - | A391C | 12% |
|  |  | G393A | 9% | G393A | 16% |
|  |  | - | - | A394G | 14% |
|  |  | - | - | A396G | 70% |
|  |  | - | - | A397G | 14% |
|  |  | - | - | G398A | 15% |
|  |  | - | - | A400G | 15% |
|  |  | - | - | A403C | 85% |
|  |  | - | - | G404C | 14% |
|  |  | - | - | A405G | 14% |
|  |  | - | - | G407A | 14% |
|  |  | - | - | A408G | 14% |
|  | | | | | |
| **ø** | 184-207,  586-630 | - | - | G185A | 15% |
|  |  | - | - | T186G | 14% |
|  |  | - | - | A187T | 14% |
|  |  | - | - | T189A | 14% |
|  |  | - | - | A190T | 14% |
|  |  | - | - | T191A | 15% |
|  |  | - | - | C192A | 85% |
|  |  | - | - | C192T | 15% |
|  |  | - | - | G195A | 100% |
|  |  | - | - | G196A | 14% |
|  |  | - | - | A197G | 14% |
|  |  | - | - | A200C | 84% |
|  |  | - | - | T201C | 98% |
|  |  | - | - | A202C | 14% |
|  |  | - | - | G204A | 99% |
|  |  | - | - | T205A | 15% |
|  |  | - | - | G206T | 14% |
|  |  | - | - | T207C | 85% |
|  |  | - | - | T207G | 15% |
|  |  | - | - | A586C | 14% |
|  |  | - | - | A588G | 84% |
|  |  | - | - | A589G | 14% |
|  |  | - | - | C591T | 78% |
|  |  | - | - | C591A | 14% |
|  |  | - | - | A593T | 15% |
|  |  | - | - | T594C | 46% |
|  |  | - | - | T594A | 14% |
|  |  | - | - | A595T | 9% |
|  |  | - | - | A595C | 6% |
|  |  | - | - | T596A | 14% |
|  |  | - | - | A597T | 14% |
|  |  | - | - | G598A | 100% |
|  |  | - | - | T600C | 19% |
|  |  | - | - | T600A | 15% |
|  |  | - | - | A601T | 14% |
|  |  | - | - | A603C | 84% |
|  |  | - | - | A605C | 15% |
|  |  | A606G | 88% | - | - |
|  |  | - | - | T607A | 14% |
|  |  | G609A | 12% | G609A | 86% |
|  |  | - | - | G609T | 14% |
|  |  | - | - | T610A | 15% |
|  |  | - | - | A612T | 14% |
|  |  | - | - | C613A | 15% |
|  |  | T615C | 34% | T615C | 81% |
|  |  | - | - | A616C | 14% |
|  |  | - | - | T617A | 15% |
|  |  | - | - | T618A | 85% |
|  |  | - | - | G619A | 14% |
|  |  | - | - | T620G | 14% |
|  |  | - | - | G621A | 85% |
|  |  | G621T | 39% | G621T | 15% |
|  |  | T624C | 97% | T624A | 14% |
|  |  | - | - | A625C | 75% |
|  |  | - | - | A625T | 14% |
|  |  | - | - | A626C | 15% |
|  |  | - | - | G627A | 100% |
|  |  | - | - | C628C | 85% |
|  |  | - | - | A629C | 14% |
|  |  | - | - | A630G | 86% |
|  | | | | | |
| **α2α3β3β4**  **(AM14)** | 442-582 | - | - | T443A | 14% |
|  |  | - | - | C444A | 85% |
|  |  | - | - | C444T | 14% |
|  |  | - | - | C446G | 14% |
|  |  | - | - | C447A | 85 |
|  |  | - | - | G449A | 14% |
|  |  | - | - | T450G | 15% |
|  |  | - | - | G451T | 14% |
|  |  | - | - | C453T | 80% |
|  |  | - | - | C453G | 14% |
|  |  | - | - | A454T | 15% |
|  |  | - | - | T455A | 15% |
|  |  | - | - | T456A | 84% |
|  |  | - | - | G457A | 14% |
|  |  | - | - | C458G | 14% |
|  |  | T459C | 44% | T459C | 14% |
|  |  | - | - | G460T | 14% |
|  |  | - | - | T461G | 14% |
|  |  | - | - | A462T | 14% |
|  |  | - | - | T463A | 14% |
|  |  | - | - | C464T | 15% |
|  |  | T465C | 65% | T465C | 91% |
|  |  | - | - | T465A | 8% |
|  |  | - | - | A466C | 14% |
|  |  | G468A | 19% | G468A | 98% |
|  |  | - | - | G469A | 14% |
|  |  | - | - | T470G | 14% |
|  |  | - | - | C472T | 16% |
|  |  | - | - | T473C | 14% |
|  |  | G474A | 13% | G474A | 85% |
|  |  | - | - | G474T | 15% |
|  |  | - | - | C475A | 14% |
|  |  | - | - | A476C | 15% |
|  |  | - | - | C477A | 15% |
|  |  | T478C | 98% | T478C | 98% |
|  |  | - | - | T479C | 14% |
|  |  | - | - | A480T | 92% |
|  |  | - | - | A480C | 6% |
|  |  | - | - | G481T | 14% |
|  |  | - | - | A482G | 14% |
|  |  | - | - | G484A | 14% |
|  |  | A486G | 95% | A486G | 14% |
|  |  | - | - | G487A | 15% |
|  |  | - | - | A488G | 14% |
|  |  | - | - | G490A | 14% |
|  |  | - | - | T491G | 14% |
|  |  | - | - | G492T | 14% |
|  |  | - | - | A493G | 14% |
|  |  | - | - | C495A | 14% |
|  |  | - | - | A496C | 14% |
|  |  | G498A | 89% | G498A | 17% |
|  |  | - | - | A499G | 14% |
|  |  | - | - | T500A | 15% |
|  |  | C501A | 6% | C501T | 14% |
|  |  | - | - | A502C | 14% |
|  |  | G506A | 8% | - | - |
|  |  | - | - | T507A | 15% |
|  |  | - | - | G508T | 14% |
|  |  | - | - | C509G | 15% |
|  |  | - | - | T510C | 17% |
|  |  | - | - | C511T | 94% |
|  |  | A513G | 14% | A513G | 85% |
|  |  | - | - | A513T | 14% |
|  |  | - | - | C514T | 55% |
|  |  | - | - | C514G | 14% |
|  |  | - | - | A516G | 73% |
|  |  | - | - | A516T | 14% |
|  |  | - | - | T517G | 12% |
|  |  | - | - | C518T | 15% |
|  |  | - | - | C519T | 84% |
|  |  | - | - | A520T | 14% |
|  |  | - | - | C521A | 14% |
|  |  | - | - | A522C | 14% |
|  |  | - | - | C525A | 14% |
|  |  | - | - | A526C | 14% |
|  |  | - | - | G528A | 97% |
|  |  | - | - | G529A | 14% |
|  |  | - | - | C530G | 14% |
|  |  | C531T | 97% | C531T | 85% |
|  |  | - | - | G532T | 14% |
|  |  | - | - | T533G | 14% |
|  |  | - | - | A534T | 14% |
|  |  | - | - | G535A | 15% |
|  |  | - | - | T536G | 14% |
|  |  | - | - | C537T | 14% |
|  |  | - | - | A538C | 14% |
|  |  | - | - | G539A | 15% |
|  |  | - | - | C540T | 85% |
|  |  | - | - | C540G | 15% |
|  |  | - | - | T541C | 86% |
|  |  | - | - | T542C | 14% |
|  |  | - | - | A543T | 14% |
|  |  | - | - | T544A | 14% |
|  |  | - | - | C545T | 14% |
|  |  | A546T | 42% | A546C | 14% |
|  |  | - | - | T549A | 15% |
|  |  | - | - | G550T | 14% |
|  |  | - | - | A552G | 98% |
|  |  | - | - | T554G | 14% |
|  |  | T555C | 44% | T555C | 78% |
|  |  | - | - | G557A | 15% |
|  |  | - | - | T558G | 14% |
|  |  | - | - | G559T | 14% |
|  |  | - | - | T560G | 14% |
|  |  | - | - | C561T | 98% |
|  |  | - | - | A564A | 14% |
|  |  | - | - | C566A | 14% |
|  |  | - | - | A568C | 14% |
|  |  | - | - | G569A | 14% |
|  |  | - | - | C570G | 15% |
|  |  | - | - | A571C | 14% |
|  |  | A573G | 19% | - | - |
|  |  | - | - | T574A | 14% |
|  |  | - | - | T575G | 14% |
|  |  | - | - | G576T | 14% |
|  |  | - | - | T577G | 14% |
|  |  | - | - | A579T | 14% |
|  |  | - | - | G580A | 14% |
|  |  | - | - | A581G | 14% |
|  |  | - | - | C582T | 81% |
|  |  | - | - | C582A | 15% |
|  | | | | | |
| **MPE8** | 130-150,  913-930 | - | - | A131T | 15% |
|  |  | - | - | T132C | 23% |
|  |  | - | - | T132A | 15% |
|  |  | - | - | C133T | 93% |
|  |  | - | - | T135G | 14% |
|  |  | - | - | A136T | 12% |
|  |  | - | - | G137A | 15% |
|  |  | - | - | T138G | 15% |
|  |  | - | - | G139T | 14% |
|  |  | - | - | C140G | 14% |
|  |  | - | - | T141C | 14% |
|  |  | C142T | 18% | C142T | 98% |
|  |  | - | - | A144T | 15% |
|  |  | - | - | A145C | 8% |
|  |  | - | - | G146A | 12% |
|  |  | - | - | A147G | 16% |
|  |  | - | - | C149A | 14% |
|  |  | - | - | T150A | 85% |
|  |  | - | - | T150C | 14% |
|  |  | - | - | C913A | 85% |
|  |  | - | - | C913T | 15% |
|  |  | - | - | T914A | 15% |
|  |  | - | - | A915C | 86% |
|  |  | - | - | A915T | 14% |
|  |  | - | - | T916C | 14% |
|  |  | - | - | A917T | 14% |
|  |  | - | - | T918A | 14% |
|  |  | - | - | G919T | 15% |
|  |  | - | - | T921G | 14% |
|  |  | - | - | G922T | 14% |
|  |  | - | - | T923G | 14% |
|  |  | - | - | G924A | 86% |
|  |  | - | - | G924T | 14% |
|  |  | - | - | T926A | 14% |
|  |  | - | - | A927T | 3-% |
|  |  | - | - | G928A | 15% |
|  |  | - | - | A929G | 14% |
|  |  | - | - | T930A | 14% |

Changes with ≤5% frequency were omitted from this table. Individual genotypes of each subgroup contributed equally to the proportion of amino acids found at each residue.
