# Supplementary figures and images for: Sequence variability of the respiratory syncytial virus (RSV) fusion gene among contemporary and historical genotypes of RSV/A and RSV/B
Source: PLoS One. 2017 Apr 17;12(4):e0175792. doi: 10.1371/journal.pone.0175792 (PMC5393888; doi:10.1371/journal.pone.0175792)

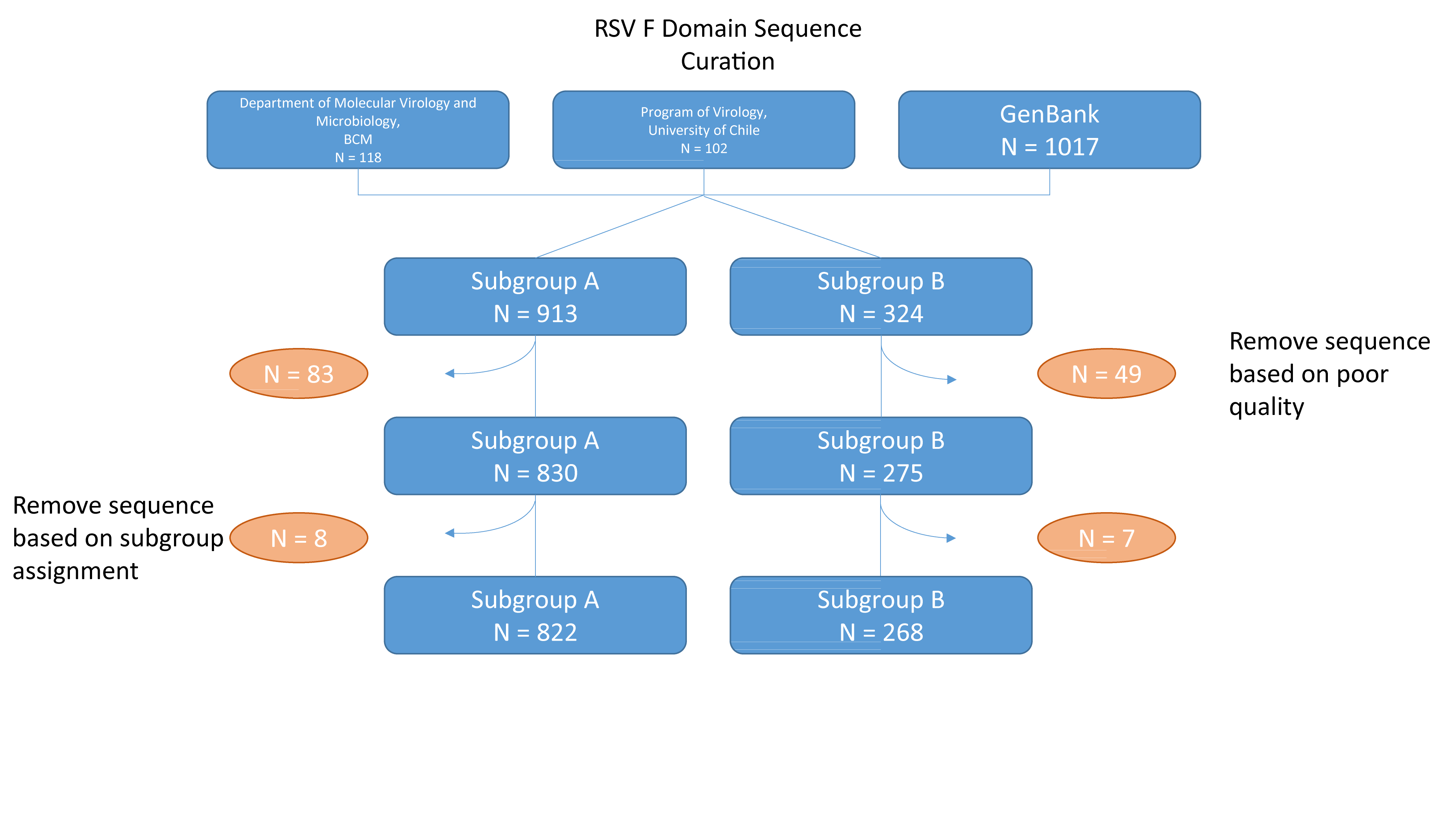

Supplement: S1 Fig — (TIF) [file pone.0175792.s004.tif]

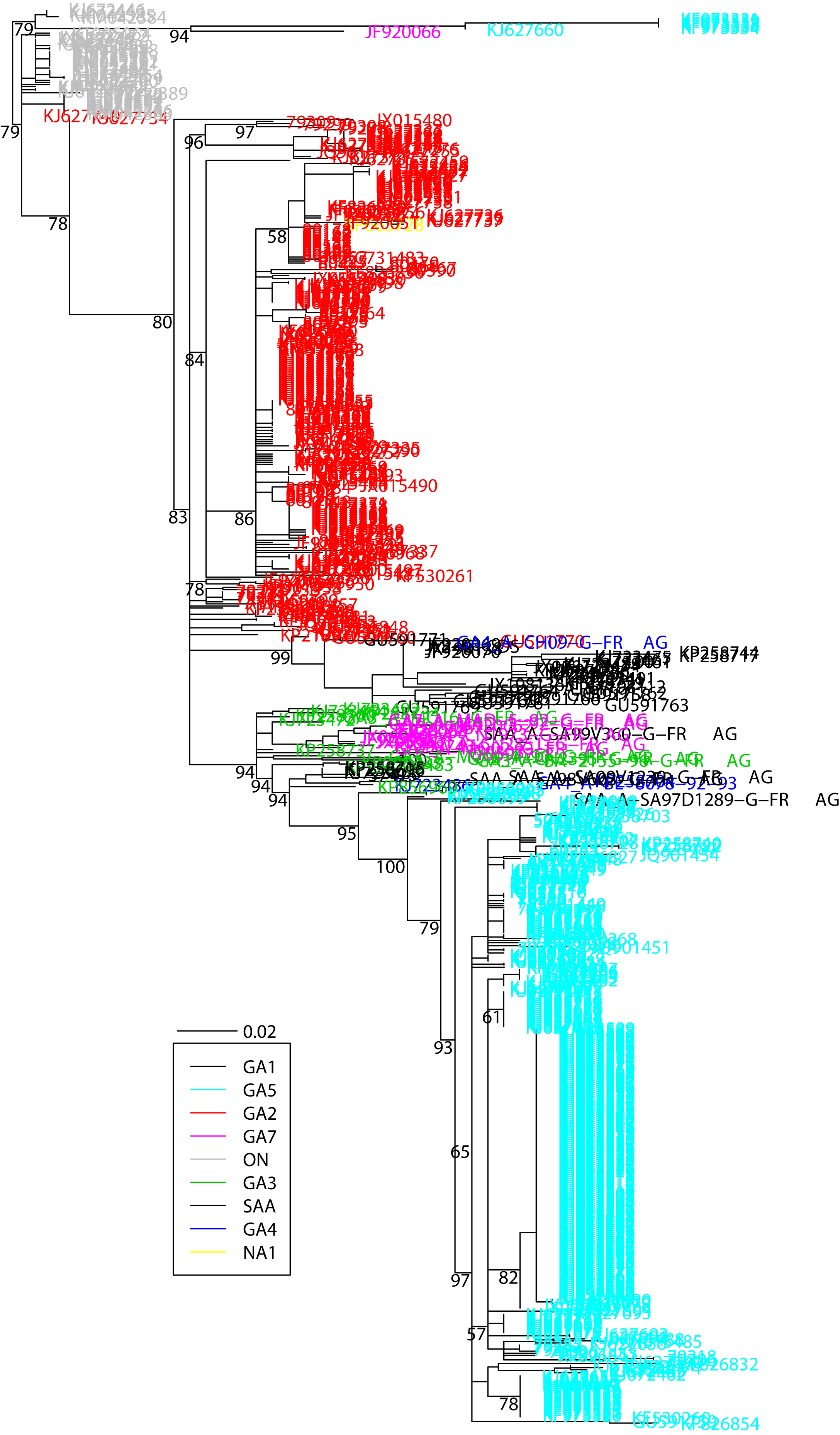

Supplement: S2 Fig — (TIF) [file pone.0175792.s005.tif]

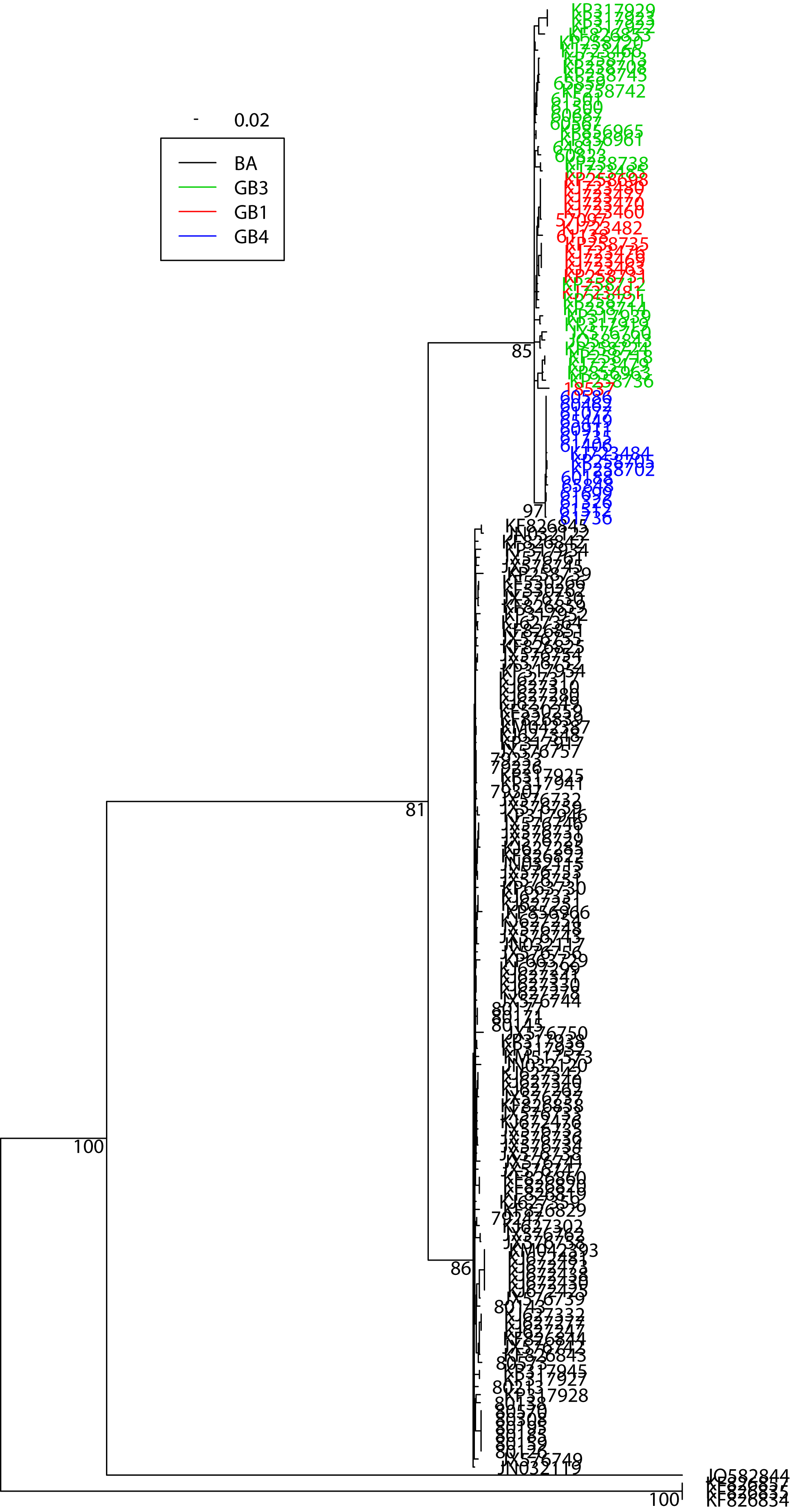

Supplement: S3 Fig — (TIF) [file pone.0175792.s006.tif]

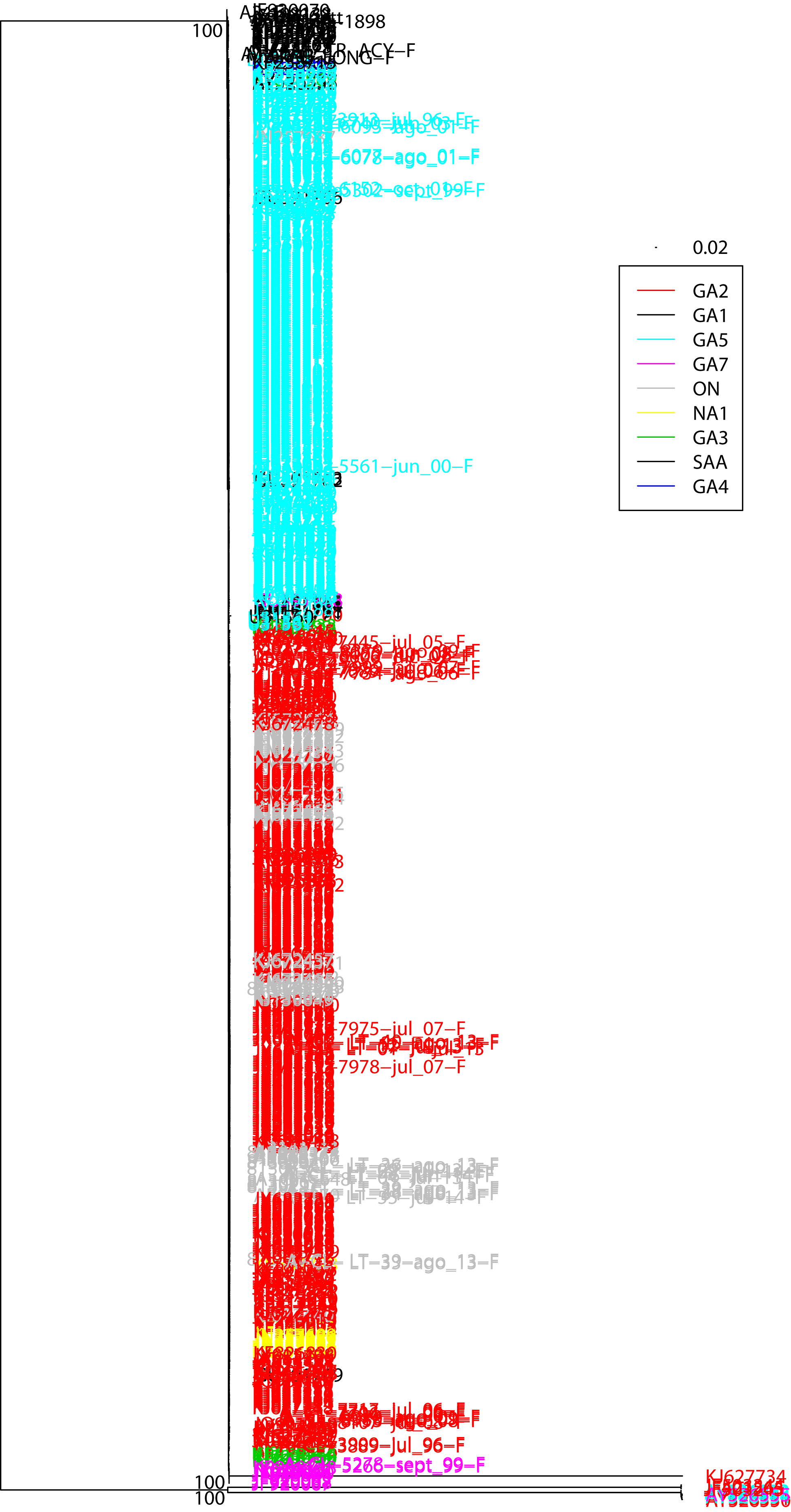

Supplement: S4 Fig — (TIF) [file pone.0175792.s007.tif]

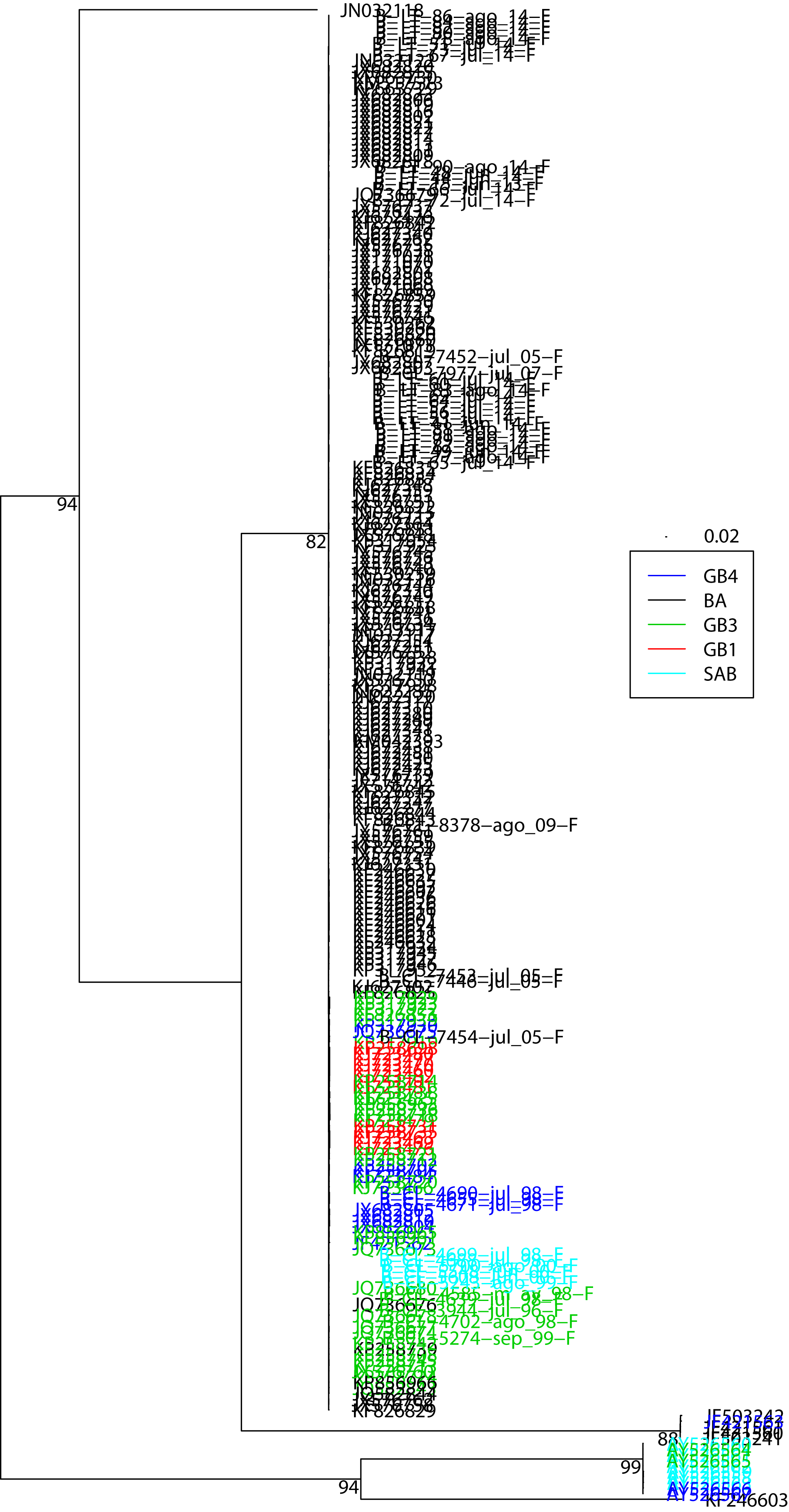

Supplement: S5 Fig — (TIF) [file pone.0175792.s008.tif]
